# Antimicrobial, antibiofilm, antioxidant activities and molecular docking of Schiff base and its complexes of Cu(II), Co(II), Ni(II), Mn(II) and UO_2_(II)

**DOI:** 10.1038/s41598-026-54902-5

**Published:** 2026-06-05

**Authors:** Reham H. Wahba, Elsayed M. AbouElleef, E. A. Gomaa, M. A. Diab, M. I. Abou-Dobara, M. M. El-Zahed, A. Z. El-Sonbati

**Affiliations:** 1 Chemistry Department, Faculty of Science, Damietta University, Damietta, Egypt; 2 Basic Sciences Department, Delta Higher Institute for Engineering and Technology, Dakhlia, Mansoura, 35681 Egypt; 3 Chemistry Department, Faculty of Science, Mansoura University, Mansoura, 35516 Egypt; 4 Botany and Microbiology Department, Faculty of Science, Damietta University, New Damietta City, 34517 Egypt

**Keywords:** Bis-Schiff base, Metal complexes, Antimicrobial, Antibiofilm, Antioxidant, Molecular docking

## Abstract

The global escalation of multidrug-resistant pathogens demands the urgent development of multifunctional metallo-pharmaceuticals. Coordination compounds offer a unique advantage by combining ligand bioactivity with metal-centered redox potential. A novel tetradentate N_2_O_2_ Schiff base ligand (H_2_L) was synthesized via the condensation of 2,6-diaminopyridine and 2,4-dihydroxybenzaldehyde. Its coordination potential was explored through the synthesis of Cu(II), Co(II), Ni(II), Mn(II), and UO_2_(II) complexes. Comprehensive characterization was performed using spectroscopic (FT-IR, NMR, UV–Vis) and physicochemical techniques. Biological efficacy was quantified through antimicrobial (MIC/MMC), antibiofilm, and DPPH radical scavenging assays, supplemented by enzymatic (POX/CAT) profiling. Molecular docking studies using MOE 2019 targeted essential proteins from *B. cereus*, *S. aureus*, *E. coli*, and *S. typhi* (PDB: 1FEZ, 3Q8U, 3T88, and 6J90). Structural analysis confirmed the tetradentate dibasic nature of H_2_L. Metal complexation significantly enhanced biological potency; the UO_2_(II) and Cu(II) complexes exhibited superior antimicrobial effects, while the Mn(II) complex demonstrated the highest antioxidant potential (78.1% inhibition; IC_50_ = 64.10 µg/mL), acting as a functional SOD-mimic. Biofilm formation in *S. aureus, B. cereus,* and *E. coli* was suppressed by up to 50% at 100 µg/mL of ligand and its complexes. Computational analysis revealed that Co(II) and UO_2_(II) complexes achieved the most favorable binding affinities, stabilized by hydrogen bonding and π-cation interactions. These findings suggest that these complexes represent promising scaffolds for next-generation antimicrobial and antioxidant agents, with significant potential for therapeutic application in disrupting microbial redox homeostasis.

**Supplementary Information:**

The online version contains supplementary material available at 10.1038/s41598-026-54902-5.

## Introduction

The increasing microbial resistance to widely exhausted antimicrobial agents is a serious therapeutic concern that has led scientists to create innovative antimicrobial agents that might be efficient against the altered infections^1,2^. A promising family of potent antibacterial drugs is the imine (–N = CH) group, which is readily created when amines combine with carbonyl compounds^3,4^. Schiff base is an excellent chelating agent owing to their C = N bond, particularly once a functional group as –SH or –OH is next to C = N. This configuration improves their chelation capabilities by enabling the formation of a 5- or 6-membered ring together with a metal ion. More research in this area is greatly desired given the versatility of Schiff bases as ligands in both biological and analytical contexts, as well as their possible economic implications^5,6^.

The Schiff laws play a well-known function in coordination chemistry by making it easier for balanced complexes to form among transition metals. These complexes are widely used in many different industries, such as the polymer industry, pigments and dyes, analytical chemistry, and coordination chemistry itself. They also have biological and catalytic properties and are used as model biomolecules in vitamins and enzymes^7,8^. The ability of aromatic Schiff bases with an ortho-OH group to experience reversible color changes in response to exposure to either temperature (thermochromism) or light (photochromism) is an interesting characteristic. Particularly in light of recent developments in optical technology, these optical properties have drawn a lot of interest.

Additionally, several of these Schiff bases have the ability to promote the synthesis of polymers^9^. Since the authors were keen to create novel Schiff base complexes that may be applied to pharmacology and luminescence probes, they synthesized and investigated complexes using Schiff bases for this work. Because of their pharmacological and biological significance, as well as their ability to form chelates with transition metals, Schiff bases are considered interesting precursors for the synthesis of novel bioactive molecules with medical utility^10–12^. Those of pyridine-based privatives in particular were identified as possible antibacterial reagents^13,14^. These are used in a range of biochemical activities to produce molecular systems with biological and therapeutic implications.

The availability of many bonding sites in heterocyclic compounds with rings, such as furan, thiophene, and pyridine, led scientists to create Schiff bases from them. Nitrogen is a heteroatom with a confined pair of electrons in the pyridine ring. As a result, a large number of ligands and their pyridine-ring-containing transition metal complexes were developed to enhance their bioactivity in diverse sectors evaluated^15–21^. However, little research has been done using diaminopyridines as ligands^22–25^. The reaction of pyridine, 2,6-diamine, and 2,4-dihydroxybenzaldehyde formed these specific Schiff bases, and the paper presents the results of their investigation. When 2,6-diaminopyridine is excited with 320 nm light, fluorescence emission is seen. Additionally, Schiff bases and the copper complexes that are formed from them exhibit increased fluorescence activity when this diamine is present. This feature is highly beneficial for some of the better scientific uses of fluorescent chemicals, such as biological imaging, DNA sequencing, monitoring drug uptake by the cell by seeing the fluorescent tag, etc.

The novel tetradentate Schiff base ligand (H_2_L) was synthesized in the present study by condensation of 2,6-diaminopyridine and 2,4-dihydroxybenzaldehyde. It was then treated with Cu(II), Co(II), Ni(II), Mn(II), and UO_2_(II) acetates to get five metal complexes. Using a variety of analytical methods, for characterized these complexes. Several heterocyclic Schiff base ligand and their transition metal complexes have been actively studied for their interesting coordination chemistry as well as their biological applications, antimicrobial activities, antioxidant activity, anticancer potential, and other medicinal applications^11,12,26–31^. These include complexes with Cu(II), Co(II), Ni(II), Zn(II), Mn(II), UO_2_(II), and Ru(II) and Schiff base ligands derived from 2-amino-3-hydroxypyridine and 2,4-dihydroxybenzaldehyde^32^. Compared to aromatic aldehydes, aliphatic aldehyde and ketone Schiff base ligands are more unstable due to the existence of effective conjugation in the ring system. The presence of a donating substituent at the ortho position aids in coordination, and the azomethine group’s nitrogen atom’s sp^2^ hybidized orbital contains a single pair of electrons that contribute significantly to coordination and provide good chelating capabilities. In an ethanolic solvent, aliphatic or aromatic aldehyde condenses with primary aliphatic or aromatic amine to form Schiff base ligands. The reaction is sped up by adding a catalytic quantity of acid^33^.

In this paper, the fabrication of a novel functional NO-donor Schiff base ligand containing a pyridine and hydroxy mediety is detailed. Numerous chelates, such as Cu(II), Co(II), Ni(II), Mn(II), and UO_2_(II) metal ions, were fabricated and spectroscopically categorized. In addition, disclosed compounds were exposed to molecular docking studies, and biological activities. Additionally, correlations between the computational data and the experimental results were established.

This work is novel where the growth inhibition behavior of crystal structures of *B. cereus* (1FEZ), *S. aureus* (3Q8U), *S. typhi* (6J90), and *E. coli* (3T88) were gained from Protein Data Bank (PDB) was investigated through molecular docking of the ligand and its metal complexes **(1–5)**. Characterization of the compounds has been done using the different techniques. The Schiff base acts as tetradentate dibasic donor, coordinating through the azomethine nitrogen, and phenolic –O groups. The prepared compounds were investigated for its antibacterial qualities against *S. typhi*, *E. coli*, *S. aureus*, and *B. cereus*, as well asantifungal action against such as *F. oxysporum*, *A. niger*, *Penicillium* sp., and *C. albicans*. The antimicrobial experiments employed the minimum inhibition concentration, minimum microbicidal concentration, and agar well diffusion method.

## Materials and instruments

### Materials and instruments

All chemicals were of analytical reagent grades and used without any further purification^34–37^. 2,6-Diaminopyridine and 2,4-dihydroxybenzaldehyde was purchased from the Aldrich Chemical Company. The used chemicals purchased from Sigma including metals of Cu(CH_3_COO)_2_⋅H_2_O, Co(CH_3_COO)_2_⋅2H_2_O, Ni(CH_3_COO)_2_.H_2_O Mn(CH_3_COO)_2_·4H_2_O and UO_2_(CH_3_COO)_2_.2H_2_O.

The ligand and its complexes were subjected to elemental analysis at Cairo University Microanalytical Center in Egypt. Standard techniques were used to ascertain the complexes metal content^38–43^. FT-IR spectra were recorded using a Perkin-Elmer 1650 spectrometer (4000–400 cm^−1^) in KBr discs. Ultraviolet–Visible (UV–Vis) spectra of the complexes were recorded in Nujol solution using a Unicom SP 8800 spectrophotometer in dimethylformamide (DMF) at room temperature. Thermal Analyzer (STA) 6000 system using thermogravimetric analysis (TGA) method. Thermal properties of the samples were analyzed in the temperature range from 30 to 800 °C at the heating rate of 10 °C/min under dynamic nitrogen atmosphere. X-Ray diffraction analysis of complexes was recorded on X-ray diffractometer analysis in the range of diffraction angle 2θ° = 4–80° with Cu K_α1_-radiation. The applied voltage and the tube current are 40 kV and 30 mA, respectively.

### Preparation of Schiff base ligand (H_2_L)

**Fig. 1:**
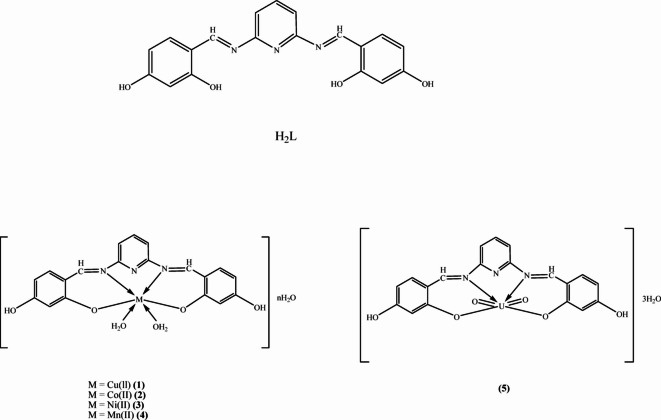
The structure of the Schiff base ligand (H_2_L) and the metal complexes.

Schiff base ligand of 4,4'-((1E,1'E)-(Pyridine-2,6-diylbis(azanylylidene)) bis(methanylylidene))bis(benzene-1,3-diol) (H_2_L) was prepared according to the previous procedure^1–3^. An ethanolic solution of 2,6-diaminopyrine (0.1 mmol) was added slowly to the solution of 2,4-dihydroxybenzaldehyde (0.1 mmol) in ethanol with constant stirring and addition two drops of piperidine. The mixture was refluxed for 4 h in a water bath. After concentration of the solution, the precipitate was separated, filtered, washed with ethanol and dried in vacuum over anhydrous CaCl_2_^4^. Recrystallization from ethanol afforded pure crystals, Yield: 88%. The purity of ligand was checked by TLC. Our synthetic route of Schiff base ligand is shown in Fig. 1.

### Preparation of the complexes

**Fig. 2:**
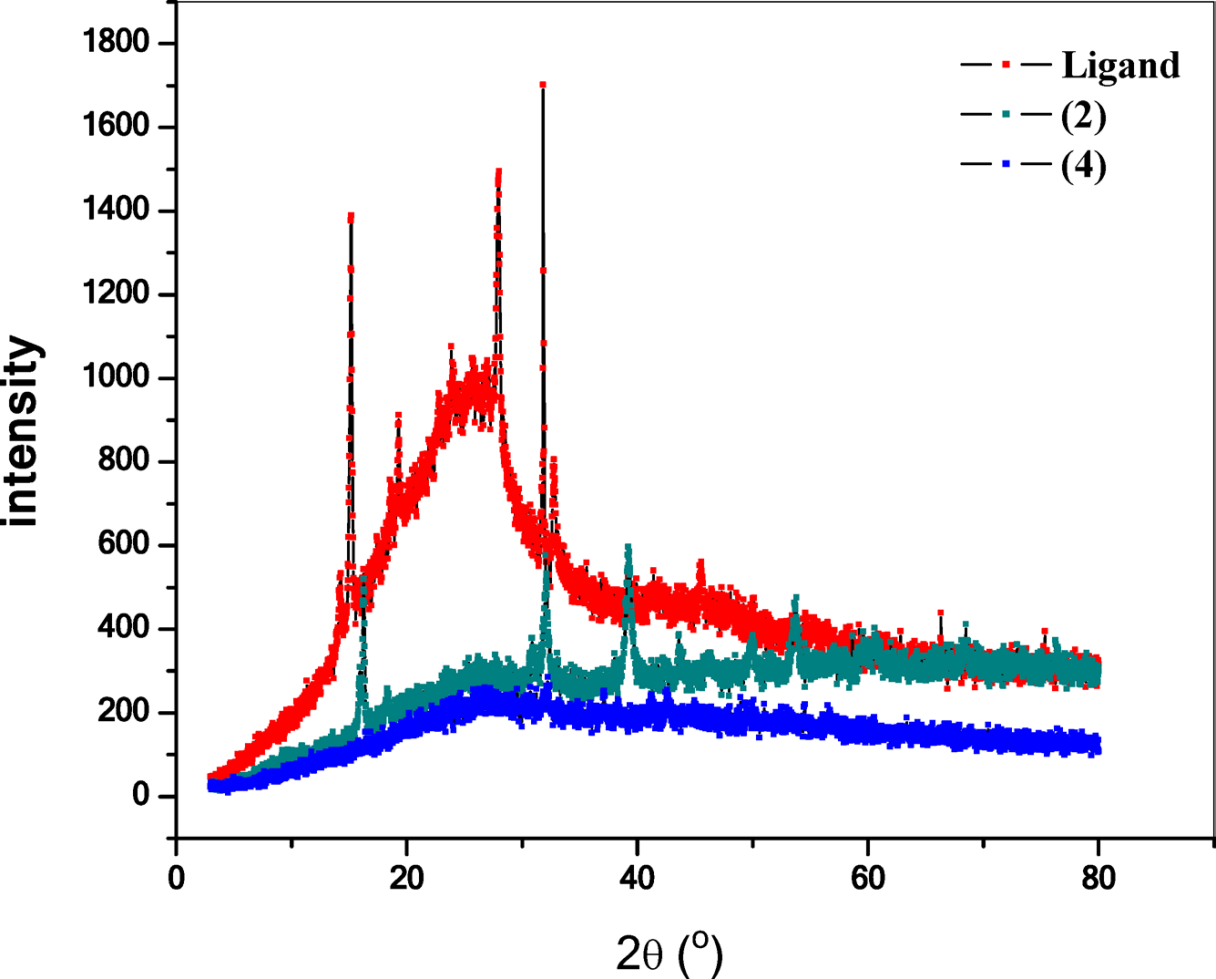
X-ray diffraction patterns of the ligand and complexes (**2**) and (**4**).

The metal complexes **(1–5)** of the different metal ions under study were prepared and purified by recrystallization when possible (Fig. 2). The following synthetic has been employed. Ethanolic solution of metal salt [Cu(II), Co(II), Ni(II), Mn(II) and UO_2_(II)] acetate (0.001 mol) was added slowly to the ligand (H_2_L) (0.001 mol) in ethanol and the reaction mixture was further refluxed. The colored precipitates were filtered through sintered glass crucible and washed several times with hot ethanol, ether and finally dried in vacuum over anhydrous calcium chloride in desiccators. The results of elemental analysis as well as some physical properties and the suggested chemical formula are listed in Table 1. The structure of the ligand and it complexes was represented in Fig. 1. Unfortunately, single crystals of the studied compounds could not be obtained because of lack of solubility in most organic solvents^2,4^.

**Table 1:** Elemental analyses data physical properties of H_2_L ligand and its complexes.

Compound	Yield (%)	M.p. (°C)	Calcd. (Found).)% μ_eff_ (B.M.)			
			C	H	N	
H_2_L	88	160	65.33 (65.19)	4.30 (4.19)	12.03 (11.89)	–
**[**CuL(H_2_O)_2_] **(1)**	80	˃300	51.06 (51.13)	3.81 (3.93)	9.41 (9.66)	1.85
[CoL(H_2_O)_2_]2H_2_O **(2)**	84	˃300	47.71 (47.84)	3.56 (3.63)	8.79 (8.97)	4.29
[[NiL(H_2_O)_2_]3H_2_O **(3)**	79	> 300	46.00 (46.22)	3.43 (3.52)	11.84 (12.23)	2.81
[MnL(H_2_O)_2_]4HO **(4)**	89	˃300	44.71 (44.84)	3.34 (3.43)	8.42 (8.68)	5.81
[UO_2_L]3H_2_O **(5)**	89	> 300	33.98 (34.12)	1.94 (2.03)	6.26 (6.35)	Dia

### Antimicrobial activity using agar well diffusion technique

Using sterile cold-molted nutrient agar or DOX agar media infected with pathogenic microbial strains (1–2 × 10^8^ CFU/mL), the compounds’ antibacterial and antifungal properties were assessed versus a range of bacteria (*B. cereus*, *S. aureus*, *E. coli*, and *S. typhi*) and fungi (*A. niger*, *F. oxysporum*, *Penicillium* sp., and *C. albicans*) strains. Once the agar plates had solidified, sterile cork-borers were used to create wells (1 cm). Each medication was made in 100 µl (50, 100, and 150 µg/mL), placed in the wells, and then incubated for either five days at 30°C (for the fungus) or twenty-four hours at 37°C (for the bacteria). The synthesized compounds were contrasted with the standards of penicillin G and miconazole. The zones of inhibition (ZOI) were calculated in mm^22,44^.

### Minimum inhibition concentration

The minimum inhibition concentration (MIC) values under investigation was studied utilizing Mueller–Hinton broth (MHB) broth or DOX broth media^45,46^. Following inoculation with 0.5 McFarland of the investigated microbial strains, compounds in a variety of dilutions (0–150 μg/mL) were generated then placed in broth flasks. For bacteria, the flasks were kept for 24 h at 37 °C, and for fungus, or cultured for 5 days at 30 °C. At a wavelength of 600 nm, the MIC values were measured using a UV–Visible Spectrophotometer.

### Minimum microbicidal concentration

Mueller–Hinton agar or DOX agar plates were treated with 0.1 ml of MICs using pour plate method and kept for 24 h at 37°C (bacteria) or 5 days at 30°C (fungus). Microbial growth was measured and recorded^47^.

### Peroxidase activity assay

The MIC value for each chemical was used to study the peroxidase (POX) activity of the examined microorganisms^48,49^. At 510 nm, the enzymatic activity was examined by spectrophotometry. Moles of H2O2 per liter was equivalent to one unit per milliliter of enzyme.

### Catalase activity assay

Catalase (CAT) activity of the tested microbes treated by the MIC value from each compound was also estimated^50^. The enzymatic activity was investigated spectrophotometrically at 415 nm.

### Antibiofilm test

The generated compounds’ antibiofilm ability was assessed versus *B. cereus*, *S. aureus*, and *E. coli*^51^. Different dilutions (50–150 µg/mL) of each chemical were investigated. The biofilm inhibitory percentage was calculated using the following formula: Antagonistic effectiveness is equal to *(A1-A2)/A1* × 100, where *A1* is optical density of control and *A2* is optical density of treated microbes.

### Antioxidant assay

The antioxidant activity via DPPH radical scavenging was investigated^52^.To compare the results, ascorbic acid was utilized as a typical antioxidant agent. Different dosages of the DPPH solution (5–100 µg/mL) were given to the samples and standards separately. Before the absorbance measurements were calculated, tests were kept for ½ hour at 25 °C. The formula *%* = *(A*_*control*_* − A*_*compound*_*/A*_*control*_*)* × 100 was used to calculate inhibition of DPPH (I%), where *A*_*control*_ represents optical density in an absence of compound while*A*_*compound*_ represents optical density in its presence.

### Molecular docking assay

The Molecular Operating Environment (MOE 2019) program was utilized to implement molecular docking investigations. Crystal structures for *B. cereus* (1FEZ), *S. aureus* (3Q8U), *S. typhi* (6J90), and *E. coli* (3T88) were gained from Protein Data Bank (PDB)^53,54^. Protonation at physiological pH, water molecule removal, and minimal energy use were used to make proteins. The site finder ultimately generated the active binding sites, which served as the binding pocket’s mimic sites.

By fine-tuning their 3D geometries and allocating partial charges using the MMFF94x force field, the ligand and its complexes with transition metals (Cu(II), Co(II), Ni(II), Mn(II), and UO_2_(II)) were able to attach onto active sites of bacterial crystal structures^55–57^.

Triangle Matcher placement approach for molecular docking was used to dock stiff receptor atoms for 100 ns. The London dG was used as a scoring function, and rescoring was done using the GBVI/WSA dG methods. For each ligand–protein pair, many postures were created, and the top five were chosen for more research. 2D and 3D interaction diagrams were created to show how both ligands attach to each protein’s active regions. These graphic tools brought attention to specific encounters. The docked complexes were examined to ascertain the interactions between the ligands under investigation and the residues of the protein’s active site^58–60^.

## Results and discussion

### Characterization of Schiff base and its complexes

The reaction of the prepared ligand with Cu(II), Ni(II), Co(II), Mn(II) and UO_2_(II) metal ions leading to the formation of complexes. The elemental analyses (C, H and N) and some physical properties of the ligand and its complexes are listed in Table 1 and show a good agreement with the calculated values and display the formation of 1:1 [M:L] ratio.

Metal(II) acetate reacts with the ligand in warm conditions and forms complexes. Precipitates were formed upon cooling, which were isolated and characterized by some conventional methods. Complexes of the Schiff base ligand were synthesized by following the reaction scheme as shown in Fig. S1. All complexes exhibit high melting points, indicating a strong bonding between the ligand and metal ion. All complexes are stable at room temperature. The yields of the purified complexes are in the range 70–85%. The complexes are not soluble in methanol, ethanol, acetone and chloroform, and soluble in dimethylformamide and dimethylsulfoxide. The preparation of the complexes and their observed analytical data indicate 1:1 metal:ligand stoichiometries. Thus the Schiff bases are seen to behave as dibasic tetradentate ONNO donor ligand in the prepared complexes.

The percent of carbon, hydrogen and nitrogen for Schiff base ligand (H_2_L) and its complexes was determined by elemental analyses. From the obtained results in Table 1, all compounds showed a good agreement with the calculated percent values.

FT-IR is a highly useful tool for determine structures when a powerful technique such as single-crystal XRD is not available. FTIR turns out to be a crucial instrument for verifying the development of metal complexes **(1–5)** and the Schiff base ligand (H_2_L) (Table 2). The synthesized ligand FT IR spectra revealed a stretching frequency of approximately 1622 cm^-1^ for υ(˃C = N)^4,36^. After complexation, this stretching frequency moved to the lower range 1620–1597 cm^-1^, suggesting that the ligand ˃C = N functional group is involved in coordination with metal ions^36,39^. By back-bonding from metal to the π* orbital of the azomethine group, coordination of azomethine N to metal ions reduces the electron density of ˃C = N^4,39^. In the other hand, the FTIR spectra of ligand proclaim a broad medium intensity bands in the ~ 2800–3137 cm^-1^ range which are assigned to the intramolecular hydrogen bonding vibration (O–H…..N)^2,5–7^, which disappeared in the spectra of the complexes designating the deprotonation of C2-O–H group and its coordination to metal ion. Following deprotonation, the C2-OH group coordination to metal ion is verified by formation of new band in at 540–634 cm^-1^ due to υ (M–O), whereas the HC = N- coordination to metal ion is supported by the bands in the range 430–566 cm^-1^ owing to υ(M–N)^7,36^. It is clear from FTIR spectra that H_2_L is uninegatively bidentately coupled to the metal ion. The azometine groups and the hydroxyl groups following deprotonation are the coordination sites of H_2_L. The band at 3300 cm^-1^ in the H_2_L due to υ(C4-OH) stretch is unaffected by coordination^4,36^.

**Table 2:** FT-IR, electronic spectral data of ligand and its octahedral complexes^a^.

Compd	FT-IR (cm^−1^)	λ_max_ (cm^−1^)	Assignments
**H2L**	υ(C = N)1622, υ(OH) 3300 ,3137, υ(C–O) 1222	37,730, 31,245, 25,640	π → π ∗ , *n* → π ∗
(**1**)	υ(C = N)1597, υ(C–O) 1228, υ(M–O) 581,υ (M–N) 446	15,195, 25,970, 33,670	2Eg → 2T2g, charge transfer
(**2**)	υ(C = N)1619, υ(C–O) 1223, υ(M–O) 551, υ(M–N) 430	10,860, 17,567, 22,825	υ1 υ2 υ3
(**3**)	υ(C = N)1619, υ(C–O) 1272, υ(M–O) 571, υ(M–N) 461	10,650, 18,817, 23,340	υ1 υ2 υ3
(**4**)	υ(C = N)1620, υ(C–O) 1224, υ(M–O) 634, υ(M–N) 566	17,006, 23,256, 25,510	6A1g → 4T1g(G) 6A1g → 4T2g(G) 6A1g → 4T1g(P
(**5**)	υ(C = N)1611, υ(C–O) 1225, υ(M–O) 540, υ(M–N) 520	15,195	^1^∑_g_ → ^3^Π_u_

^a^Numbers as given in Table 1 and Fig. 1

The electronic spectra of the complexes are summarized in Table 2 together with the proposed assignments and suggested geometries. The results obtained are in good agreement with other spectra and the literature^4,36,39^.

Octahedral complexes are formed by the coordination of dibasic tetra-dentate to the M(II) ion in ONNO donor Schiff base ligand fashion (Table 2). The octahedral coordination sphere in all complexes (**1–4**) is expected to be completed by the coordination of water molecules. Conductivity measurements of complexes in DMSO (10^–3^ M) indicate that all the complexes are nonelectrolytes in DMSO. The magnetic moments of complexes (**1–5**) estimated from magnetic susceptibility results as shown in Table 2.

Single crystals of the ligand (H_2_L) and its metal complexes could not be prepared to get the X-ray diffraction (XRD) and hence the powder diffraction data were obtained for structural characterization. Structure determination by X-ray powder diffraction data has gone through a recent surge since it has become important to get to the structural information of materials, which do not yield good quality single crystals.

The X-ray diffraction analysis of the ligand (H_2_L) and its complexes **(2)**, and **(4)** were shown in Fig. 2. The ligand and its complexes show multiple diffraction peaks that indicate the polycrystalline phases^17,28^.

The thermogravimetric analysis (TGA) of ligand and its complexes **(1–5)** were studied and the ass percentage loss is shown in Table S1. Complexes **(1)** and **(2)** show loss of weight corresponding to two water molecules and the complexes **(3)** and **(5)** show loss of weight corresponding to three water molecules, while Mn(II) complex **(4)** shows loss of weight corresponding to four water molecules at range of temperature 100–130 °C^4,7^.

### Agar well diffusion assessment

Microbicidal effectiveness of compounds against both Gram-positive (G + ve) and Gram-negative (G-ve) bacteria and fungi was assessed using the agar well diffusion assay. For each analyzed sample, the average ZOI width of inhibitive growth of bacterial or fungal types around wells was displayed in millimeters (Tables 3, 4 and Fig. S1). In comparison to the typical antibacterial agent, which has no inhibitory action against *S. typhi*, all complexes demonstrated strong antibacterial activity against the bacteria. Mn(II) complex exhibits the least amount of antibacterial action. *B. cereus* was unaffected by low Mn(II) complex concentrations. While less active against G + ve bacteria, all complexes were more efficient against G-ve bacteria. Cu(II) and UO_2_(II) complexes had a stronger antifungal effect against tested fungi. Miconazole was less effective against *F. oxysporum*, *A. niger*, and *Penicillium* sp. than ligand and UO_2_(II) combination. UO_2_(II) complex showed the best antifungal action against the tested fungi.

**Table 3:** Antibacterial activity of ligands and their metal mixed ligand complexes.

Ligands	Concentration, µg/mL	Gram-positive bacteria		Gram-negative bacteria	
		*Bacillus cereus*	*Staphylococcus aureus*	*Escherichia coli*	*Salmonella typhi*
Ligand	50	10 ± 0	13 ± 0	15 ± 0	14 ± 0
	100	12 ± 0	15 ± 0	17 ± 0	16 ± 0
	150	14 ± 0	17 ± 0	19 ± 0	18 ± 0
Cu(II)	50	6 ± 0.14	9 ± 0	10 ± 0.03	6 ± 0.14
	100	8 ± 0.14	11 ± 0	11 ± 0.03	8 ± 0.14
	150	10 ± 0.14	13 ± 0	13 ± 0	10 ± 0.14
Co(II)	50	8 ± 0.14	11 ± 0	11 ± 0.03	15 ± 0
	100	10 ± 0.14	13 ± 0	13 ± 0	17 ± 0
	150	12 ± 0.14	15 ± 0	15 ± 0	19 ± 0
Ni(II)	50	6 ± 0.14	9 ± 0.14	10 ± 0.03	12 ± 0.03
	100	7 ± 0.14	11 ± 0.03	12 ± 0.03	14 ± 0.03
	150	9 ± 0.14	13 ± 0.03	14 ± 0.03	16 ± 0.03
Mn(II)	50	0	10 ± 0.14	8 ± 0.14	10 ± 0.03
	100	7 ± 0.14	12 ± 0.14	11 ± 0.14	12 ± 0.03
	150	9 ± 0.14	14 ± 0.03	14 ± 0.03	14 ± 0.03
UO_2_(II)	50	8 ± 0.14	12 ± 0	11 ± 0	11 ± 0
	100	10 ± 0.06	14 ± 0	13 ± 0	13 ± 0
	150	12 ± 0.06	16 ± 0	15 ± 0	15 ± 0
Penicillin G	50	8 ± 0	10 ± 0.03	9 ± 0.14	0
	100	10 ± 0	12 ± 0.03	10 ± 0.06	0
	150	13 ± 0	16 ± 0.03	12 ± 0.06	0

**Table 4:** Antifungal activity of ligands and their metal mixed ligand complexes.

Antifungal agent	Concentration, µg/mL	Fungi			
		*Aspergillus niger*	*Fusarium oxysporum*	*Candida albicans*	*Penicillium* sp.
Ligand	50	15 ± 0.03	11 ± 0.03	13 ± 0	12 ± 0.14
	100	17 ± 0.03	13 ± 0.03	15 ± 0	14 ± 0.03
	150	19 ± 0.03	15 ± 0.03	17 ± 0	16 ± 0.03
Cu(II)	50	9 ± 0.14	12 ± 0.03	9 ± 0	-ve
	100	11 ± 0.14	14 ± 0.03	11 ± 0	6 ± 0.14
	150	13 ± 0.14	16 ± 0.03	13 ± 0	9 ± 0.14
Co(II)	50	11 ± 0.14	13 ± 0.03	11 ± 0	-ve
	100	13 ± 0.06	15 ± 0.03	13 ± 0	-ve
	150	15 ± 0.06	17 ± 0.03	15 ± 0	-ve
Ni(II)	50	12 ± 0.06	12 ± 0.14	7 ± 0.03	-ve
	100	15 ± 0.03	14 ± 0.14	10 ± 0.03	-ve
	150	17 ± 0.03	16 ± 0.14	12 ± 0	-ve
Mn(II)	50	11 ± 0.06	15 ± 0.03	9 ± 0.03	7 ± 0.14
	100	14 ± 0.03	17 ± 0.03	11 ± 0.03	9 ± 0.14
	150	16 ± 0.03	19 ± 0.03	13 ± 0.03	11 ± 0.14
UO_2_(II)	50	14 ± 0.03	13 ± 0.14	10 ± 0	14 ± 0.14
	100	16 ± 0.03	15 ± 0.03	12 ± 0	16 ± 0.03
	150	18 ± 0.03	17 ± 0.03	14 ± 0	18 ± 0.03
Miconazole	50	10 ± 0.14	10 ± 0.03	8 ± 0.03	9 ± 0.14
	100	12 ± 0.06	12 ± 0.03	11 ± 0.03	11 ± 0.14
	150	16 ± 0.03	14 ± 0.03	15 ± 0	14 ± 0.03

Conclusions show potent antibacterial behaviors of compounds and are in line with earlier research. The metal complexes outperformed the parent Schiff base ligand against one or more bacterial species^61^. The ZOI varied from 9 ± 0 mm to 35 ± 0 mm^22^. The biological activity of 4-amino antipyrines and their complexes against a range of species, including *A. niger*, *S. aureus*, *E. coli* and *K. pneumoniae*^26,62^.

Schiff base ligand’s metal complex analogues display distinctive series of MICs based on the type of metal, according to a thorough review of the data. When Schiff base bind amines or carbonyl complexes with azomethine (> C = N–), he invented Schiff bases, which are distinguished as flexible pharmacophores for pattern and elaboration of a range of biologically effective medications^63,64^. These compounds’ double bond between carbon and nitrogen has been found to be essential to their biological characteristics^65^. Numerous biological processes and chelating qualities are exhibited by these adaptable ligands. Because of the double bond’s electron-donating properties, the nitrogen atom’s lone pair of electrons, and nitrogen’s low electronegativity, nitrogen of > C = N acts as a proficient donor site. Because of the distinguishing, Schiff base is bio-active chemicals that can fight off bacterial and fungal infections, free radicals, and cancer^66,67^. These compounds’ special qualities are influenced by their binding strategies and chelating capacities toward core metal atom. Additionally, in recent years, the significance of free oxygen radicals has been highlighted. The positive effects of reactive oxygen species (ROS) are demonstrated by bio-activity of cellular reactions to anoxia, that operate as a protection versus virulent illnesses. Regardless of this benefit, novel study proposes that the oxidative damage and mutations are caused by the radicals produced throughout bio-organic oxidation–reduction manners^68,69^. Because Schiff base ligands have distinct donor atoms besides methods of attaching to metal ions, they display a broad variety of bio-functions. Through substituent modification, that modify final donating atoms, these interfaces yield a fascinating set of ligands with adjustable properties that might be used to create various compounds. Noticeable functional groups on benzene ring specially generated bioactivity versus bacteria at low concentrations, showing enhanced efficacy against *E. coli*, *P. aeruginosa, B. cereus*, *S. aureus*, *A. niger*, *C. albicans*, and *F. oxysporum*. Hydrogen bond creation through active centers in cell components with > CH = N– may hinder normal cell functions, which may be related to interruption of cell wall construction^70,71^.

### Quantitative potency analysis using activity index

To provide a rigorous analytical benchmark of the synthesized compounds against established pharmaceuticals, the activity index (AI) was calculated using the formula:

$$AI \left(\%\right)= \frac{Zone\, of\, inhibition\, of\, test\, compound\, (\hbox{mm})}{Zone\, of\, inhibition\, of\, standard\, drug\, (\hbox{mm})} \times 100$$

As illustrated in Table 5, the coordination of the Schiff base ligand to metal centers significantly modifies its pharmacological profile. Analytically, the UO_2_(II) and Co(II) complexes demonstrated exceptional efficiency. The UO_2_(II) complex achieved an AI of 100% against *S. aureus*, matching the effectiveness of the commercial drug penicillin G and 128.6% against *Penicillium sp.* relative to the standard miconazole. Most notably, several complexes—including Co(II) and UO_2_(II)—exhibited higher ZOI than penicillin G against Gram-negative *E. coli* and *S. typhi*.

**Table 5:** Activity index (%) of the ligand and its metal complexes compared to standard drugs.

Compound	*B. cereus*	*S. aureus*	*E. coli*	*A. niger*	*F. oxysporum*	*C. albicans*	*Penicillium* sp.
Standard*	100.0	100.0	100.0	100.0	100.0	100.0	100.0
Ligand	107.7	106.3	158.3	118.8	107.1	113.3	114.3
Cu(II)	76.9	81.3	108.3	81.3	114.3	86.7	64.3
Co(II)	92.3	93.8	125.0	93.8	121.4	100.0	–
Ni(II)	69.2	81.3	116.7	106.3	114.3	80.0	–
Mn(II)	69.2	87.5	116.7	100.0	135.7	86.7	78.6
UO_2_(II)	92.3	100.0	125.0	112.5	121.4	93.3	128.6

*Standards are penicillin G (bacteria) and miconazole (fungi). AI (%) = (Zone of sample/zone of standard) × 100.

### MIC and MMC studies

**Fig. 3:**
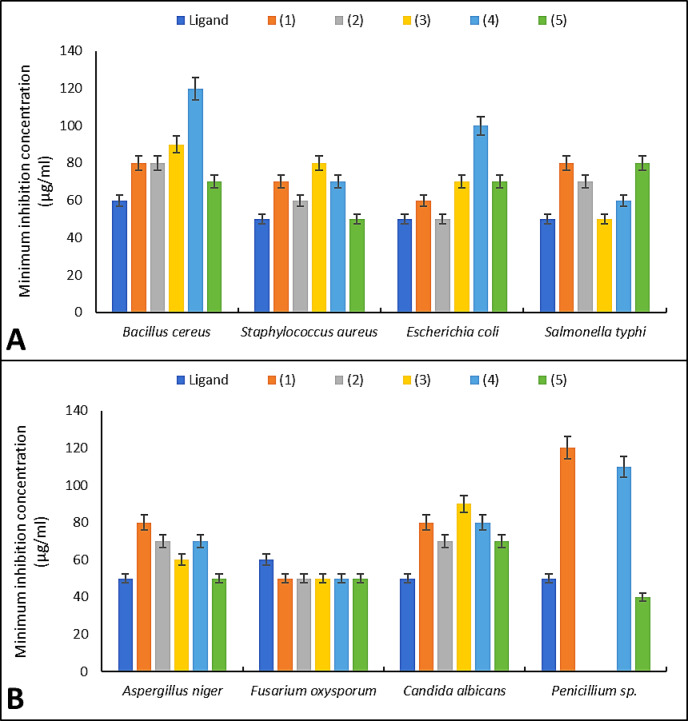
Minimum inhibition concentration of the prepared compounds against the tested bacteria; (**A**), and fungi; (**B**).

**Fig. 4:**
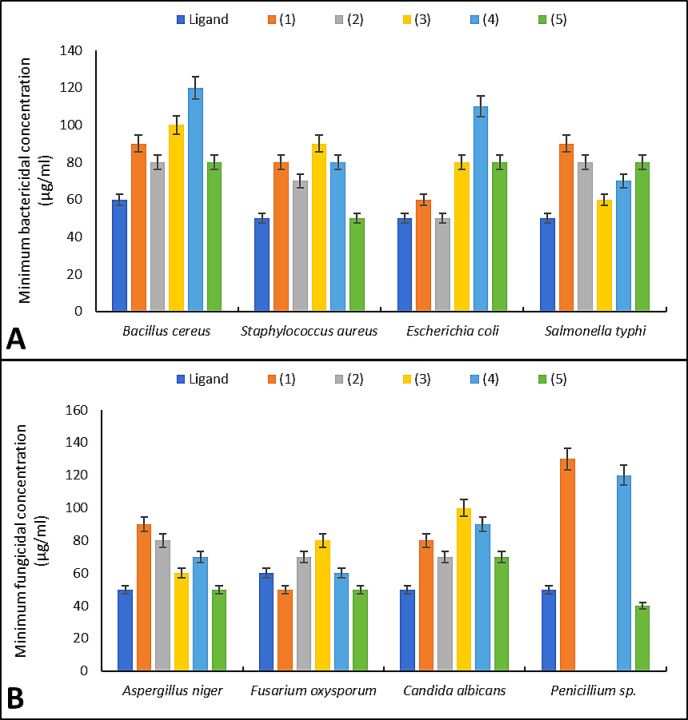
Minimum microbicidal concentration of the prepared compounds against the tested bacteria; (**A**), and fungi; (**B**).

Schiff base ligand and complexes were evaluated versus fungi, G + ve bacteria, and G-ve bacteria (Figs. 3, 4). The MIC value is the concentration at which there is complete inhibition (no detectable microbiological growth). Each chemical under investigation had a greater biocidal effect as its concentration rose^72^. At a dosage of 50–80 µg/mL, Schiff base ligand and complex (3) completely inhibited most microbial strains, demonstrating strong antibacterial and antifungal activities in contrast to other drugs. Against *E. coli*, the ligand and Co(II) complex had the greatest antibacterial action, afterward Cu(II), UO_2_(II), Ni(II), and Mn(II) complexes. MIC of Schiff base ligand and UO_2_(II) complex versus *S. aureus* and *A. niger* was matching. A. niger and *C. albicans* were suppressed by 90 µg/mL of all complexes that were generated. The *B. cereus* under investigation was only moderately to moderately susceptible to the antibacterial action of metal complexes at high doses (≥ 120 µg/mL). MMC was the lowest dose that stopped bacteria from developing on the cultured. The 4-antipyrine and its metal complexes had excellent to medium action, with MIC and MMC of around 60 µg/mL and 512 µg/mL, correspondingly^73^. 4-amino antipyrine and complexes had MIC values oscillating from 20 μg/mL to 55 μg/mL^26,74^. This high efficacy at low concentrations is supported by recent literature. The coordination of 3d-transition metals to N,O-donor Schiff bases drastically reduces the MIC by stabilizing the bioactive conformation of the ligand, allowing for more effective interaction with microbial DNA and cell-wall proteins^17^. Furthermore, the low MIC/MMC ratio (typically ≤ 4) observed for our complexes suggests a bactericidal/fungicidal rather than a bacteriostatic mechanism. This transition from inhibition to cell death is a critical hallmark of effective coordination therapeutics, as highlighted in the recent development of triaminepyrimidine-derived complexes^75^. The increased lipophilicity and structural rigidity of the chelate ring likely prevent the pathogen from utilizing efflux pumps to expel the drug, thereby ensuring sustained intracellular concentrations and lethal metabolic disruption.

The antimicrobial efficacy of the synthesized compounds was evaluated analytically by correlating the structural features with the observed MIC and MMC. The metal complexes generally exhibited enhanced potency compared to the free Schiff base ligand. This enhancement is consistently explained by Overtone’s cell permeability concept and Tweedy’s chelation theory^76,77^. Upon chelation, the polarity of the metal ion is significantly reduced due to the partial sharing of its positive charge with the donor groups (N_2_O_2_) of the ligand and the delocalization of π-electrons over the entire chelate ring. This increases the lipophilic character of the central metal atom, facilitating its passage through the lipid layers of the microbial cell membrane. While the ligand showed moderate activity, the UO_2_(II) and Cu(II) complexes demonstrated superior inhibitory effects, in some cases approaching the efficacy of the commercial standards penicillin G and miconazole. Specifically, the UO_2_(II) complex exhibited a MIC of 40 µg/ml against *Penicillium* sp., which is highly competitive when benchmarked against miconazole (80 µg/ml). The analytical data confirms that the bioactivity is not merely a function of the metal or the ligand alone, but a synergistic result of the coordination geometry and the electron-withdrawing nature of the substituents on the phenyl ring, which disrupts the respiratory chain and protein synthesis of the pathogens more effectively than the parent ligand^78,79^.

### POX and CAT activities

**Fig. 5:**
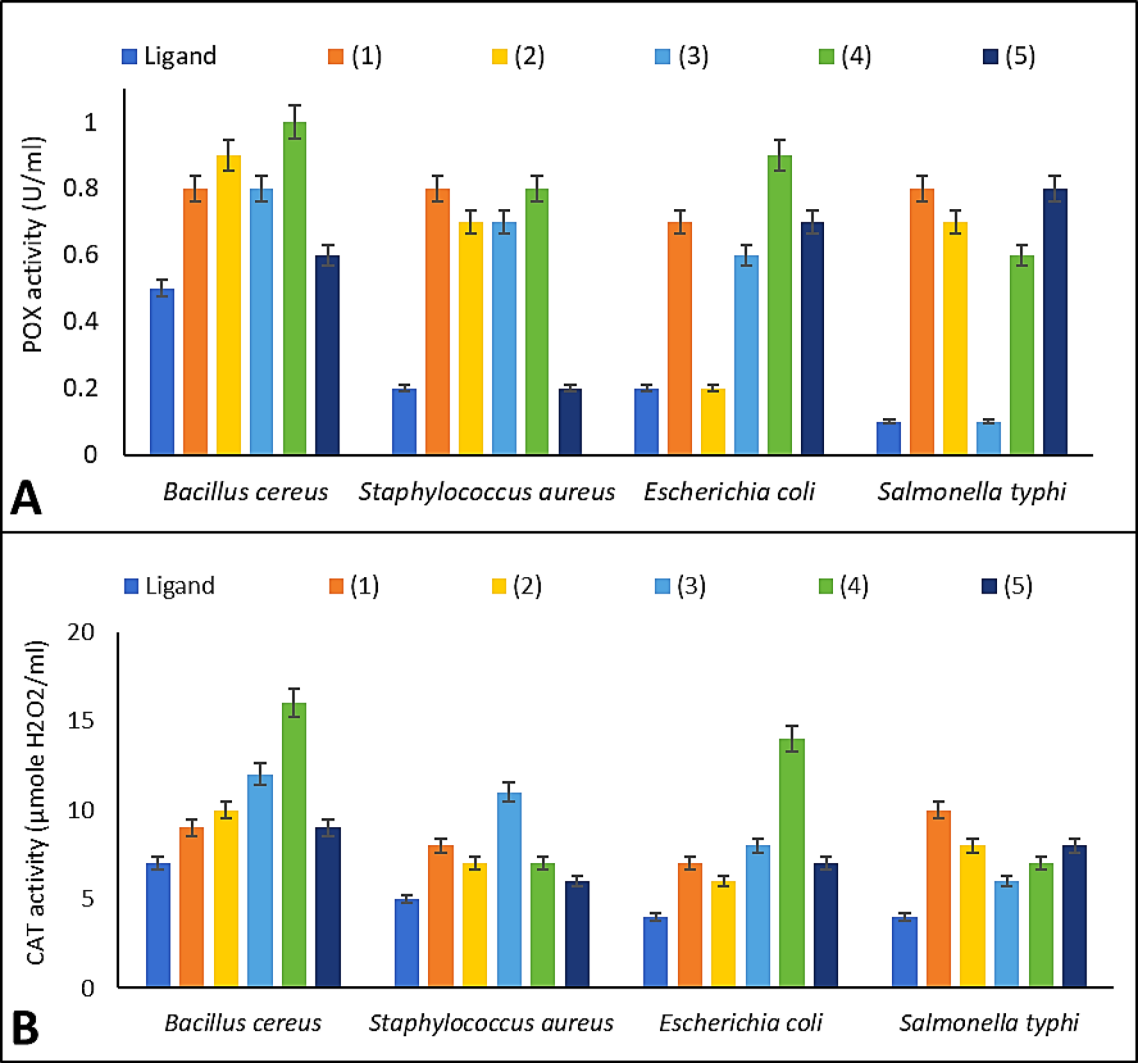
Peroxidase (POX) activity assay; (**A**), and catalase (CAT) activity assay; (**B**), of the prepared compounds.

As demonstrated by their higher CAT and POX activities compared to G-ve bacteria, the prepared compounds’ antioxidant capabilities (POX and CAT) were assessed in various bacteria (Fig. 5). Following treatment with ligand, Cu(II), or UO_2_(II) complexes, the bacteria’s POX and CAT activity ranged from mild to moderate when compared to other metal complexes, which may be owing to different cell wall constitutions of bacterial species^44,80^. Large amounts of peptidoglycan reinforce thick, solid cell wall structure of G + ve bacteria and may also make it harder for chemicals to interact with the bacterial enzymes and pass through the cell wall. The active functional groups of compounds are reliable for breaking down the bacterial cell wall and can interfere with the production of bacterial enzymes^5,81^.

### Antibiofilm properties

**Fig. 6:**
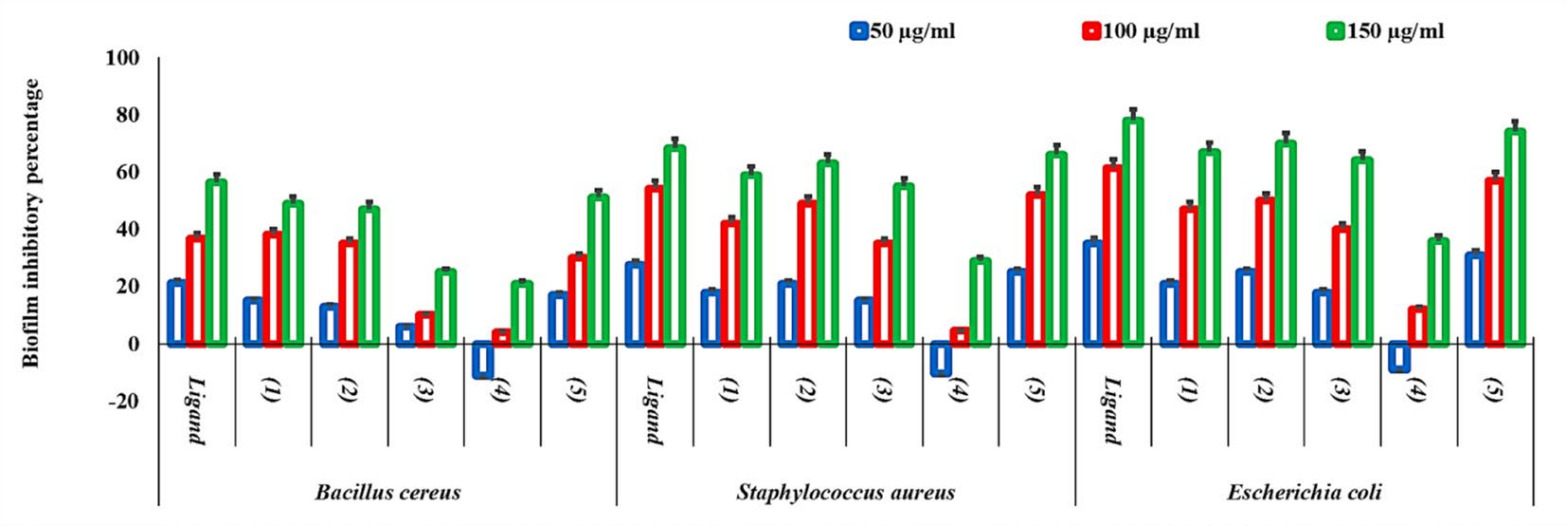
Antibiofilm assay of the prepared compounds against *B. cereus*, *S. aureus* and *E. coli*.

The antibiofilm evaluation revealed that while most complexes, particularly UO_2_(II) and Cu(II), significantly suppressed biofilm architecture, the Mn(II) complex exhibited a comparatively lower inhibition rate, and in specific instances, sustained biofilm density (Fig. 6). This observation can be analytically attributed to the biological role of manganese as an essential trace element and cofactor for various microbial enzymes. Manganese ions are known to play a critical role in the regulation of several metabolic pathways and oxidative stress response systems (such as Mn-superoxide dismutase) that protect the biofilm matrix from environmental stressors^82,83^. The inclusion of Mn(II) in the coordination sphere may partially supplement the intracellular requirement for this transition metal, potentially stabilizing microbial adhesion mechanisms rather than disrupting them. Unlike the Cu(II) or UO_2_(II) complexes, which exert high toxicity by disrupting cell membrane integrity, the Mn(II) complex likely follows a different mechanistic path where the toxicity of the chelate is partially offset by the biological utility of the metal center, leading to the observed variations in biofilm suppression. Other study reported that methicillin or any other disinfectant at sub-inhibitory dosages can dramatically accelerate the production of *S. aureus* biofilms by up-regulating the genes that encode surface proteins that are essential to the biofilm formation process^84,85^. Additionally, necessary microelements like Mn or Se encourage the microorganisms that form biofilms rather than preventing them^86^. The bacteria in our investigation used Mn at its lowest concentration (50 µg/ml) to control a range of biological activities.

The inclusion of Mn(II) in the coordination sphere likely provides a localized source of this essential trace element, which may partially counteract the toxic effects of the Schiff base ligand by supporting the microorganism’s inherent biofilm-forming pathways. This dual nature—where the toxicity of the chelate competes with the biological utility of the Mn(II) center—explains the observed persistence of biofilm density in Mn-treated samples compared to the high-toxicity profiles of Cu(II) and UO_2_(II), which lack such biological stabilization roles. Metal complexes significantly reduced the growth of *P. aeruginosa* biofilms by up to 50% at 1.7 µg/ml, and Cu(II) complexes reduced the growth of methicillin-resistant *S. aureus* biofilms by up to 60% at 25 μg/ml^87^. Similarly, copper-zinc metal complexes, through membrane damage and quorum sensing suppression, have bactericidal and antibiofilm activity versus *S. aureus* and *P. aeruginosa* at 1000 µg/ml^88^.

### Antioxidant assay

**Fig. 7:**
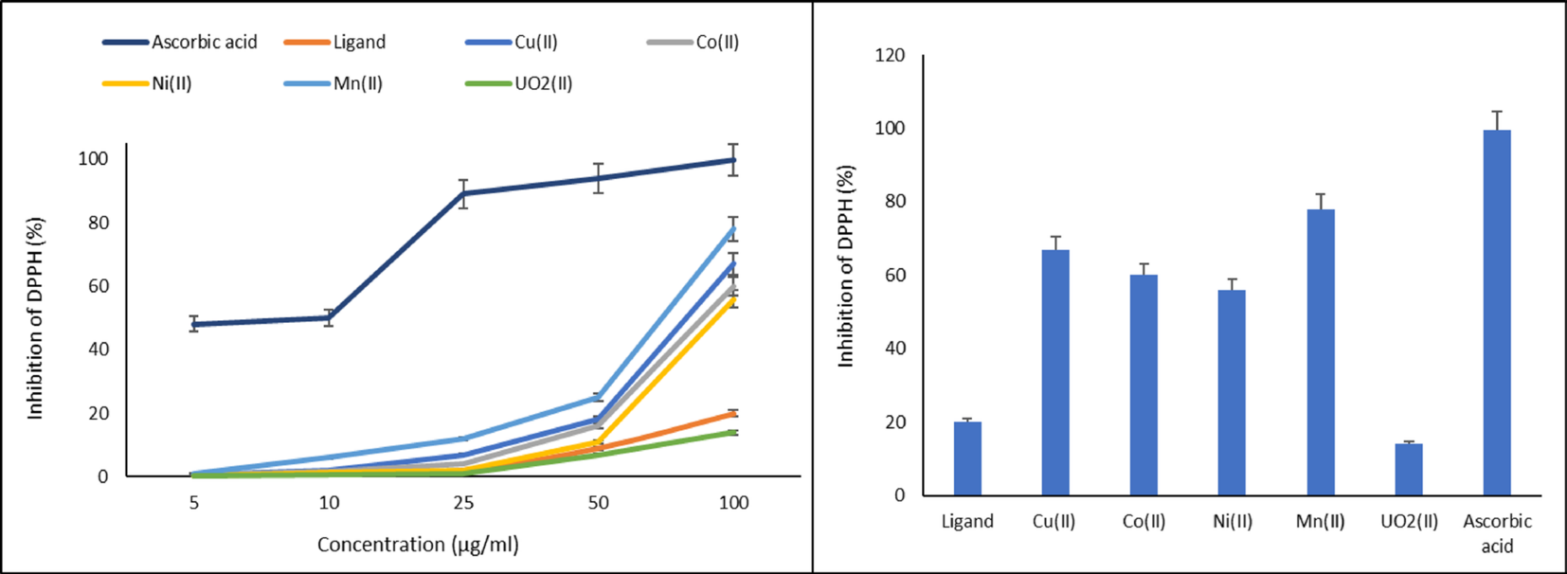
DPPH scavenging activity of different concentrations Schiff base ligand and its metal complexes compared to the standard ascorbic acid.

The antioxidant properties of the Schiff base and complexes were assessed by means of DPPH radical scavenging technique (Fig. 7). While the ABTS assay is often cited for its high sensitivity in aqueous systems, the DPPH scavenging assay was selected for this study due to its exceptional stability in organic solvents, which are necessary to maintain the solubility of coordination complexes. DPPH provides a reliable benchmark for radical quenching specifically involving hydrogen atom transfer and single electron transfer mechanisms, which are critical for assessing the redox behavior of transition metal chelates. As the concentrations of studied range grew, radical scavenging ratio increased, demonstrating that scavenging activity of compounds was concentration-dependent. Schiff base demonstrated 20.4, 67.2, 60.6, 56.6, 78.1, and 14.7% for Cu(II), Co(II), Ni(II), and UO_2_(II) complexes. At 100 µg/mL, UO_2_(II) complex had the lowest free radical scavenging activity (14.7%), while Mn(II) complex had the highest (78.1%). At all concentrations, ascorbic acid, a common antioxidant, performed better than the other substances in this study. Additionally, investigational outcomes strengthen the before published studies^89–91^.

The determination of antioxidant potential is essential to evaluate the compounds’ ability to mitigate oxidative stress—a key pathway in both antimicrobial action and host-tissue protection. The enhanced activity of the Mn(II) complex (78.1%) compared to the parent ligand can be attributed to its role as a superoxide Dismutase (SOD) mimic. Unlike the Ca, Mg, and Sr complexes of piroxicam or meloxicam reported in previous literature, which often show negligible activity due to the lack of redox-active d-orbitals, the used transition metal complexes benefit from the variable oxidation states and the stabilizing N_2_O_2_ coordination sphere^92–95^. Conversely, the UO_2_(II) complex exhibited lower inhibition than the free ligand. This behavior is likely due to the high coordination number and steric bulk of the Uranyl center, which may shield the bioactive azomethine groups from interacting with the DPPH radical. Furthermore, ascorbic acid served as a standardized positive control; its 99.6% inhibition validates the experimental setup’s sensitivity and ensures that the observed lower values for the complexes are a true reflection of their chemical structure rather than an assay limitation.

To move beyond a single-concentration description, IC_50_ (the concentration required to inhibit 50% of DPPH radicals) values were determined to provide a standardized metric of potency (Table 6). The results reveal that the Mn(II) complex possesses the highest antioxidant capacity among the synthesized compounds, with an IC_50_ of 64.10 µg/ml, significantly outperforming the free ligand (IC_50_ = 250.00 µg/ml). Analytically, the superior activity of the Mn(II) complex is attributed to its role as a SOD mimic. Manganese ions within the N_2_O_2_ coordination environment facilitate efficient radical neutralization through concerted electron transfer. The stabilization provided by the tetradentate Schiff base lowers the oxidation potential of the Mn center, allowing for easier hydrogen atom abstraction and subsequent stabilization of the DPPH radical. Furthermore, the presence of the manganese center promotes the delocalization of the unpaired electron across the chelate ring, a feature absents in the free ligand, thereby explaining the significant jump in antioxidant efficiency upon coordination.

**Table 6:** IC_50_ values of the prepared compounds for DPPH radical scavenging activity compared to ascorbic acid.

Compound	IC_50_ (µg/ml)
Ascorbic acid (standard)	50.20
Ligand	250.00
Cu(II)	74.63
Co(II)	83.33
Ni(II)	89.29
Mn(II)	64.10
UO_2_(II)	357.14

The observed differences in the generated compounds’ scavenging abilities versus DPPH radical, which illustrate the low antioxidant action, may be explained by the alterable interaction between compounds and DPPH with hydroxyl groups, as phenols. Even though compounds’ results in DPPH free radical-scavenging assay were lower than ascorbic acid, the moderate inhibition of the DPPH radical indicates that the analogues of the metal complexes of the prepared Schiff base ligand have a strong ability to scavenge free radicals. Antibacterial, antifungal, and antioxidant tests partially show the structure–activity link of the generated compounds. The greater activity and known biological properties of bio-active compounds may be due to hydroxyl and > C = N groups, conditional kind of substituent. Complexes may be a useful source for improving chemotherapeutical drugs to treat uncontrolled illnesses.

### Molecular docking study

The integration of molecular docking simulations with in vitro antimicrobial assays provides mechanistic insight into the observed biological activity of the ligand and its metal complexes. The molecular docking results were interpreted cautiously in accordance with established guidelines for structure-based drug design. Although the ligand exhibited relatively favorable docking scores against proteins from Bacillus cereus, Staphylococcus aureus, Escherichia coli, and Salmonella typhi, these values were considered as indicative of binding affinity trends rather than definitive predictors of antimicrobial activity, as recommended in previous studies^59,60^.

In the present study, docking was performed against key bacterial targets from *B. cereus* (PDB: 1FEZ), *S. aureus* (PDB: 3Q8U), *E. coli* (PDB: 3T88), and *S. typhi* (PDB: 6J90), and the results were compared with ZOI diameters obtained experimentally. The proteins chosen for the molecular docking investigation are appropriate models for predicting antimicrobial potential since they represent crucial and functionally confirmed targets in clinically relevant Gram-positive and Gram-negative bacteria. The crystal structure 1FEZ from *B. cereus* corresponds to a vital bacterial enzyme involved in core metabolic and structural processes, and inhibition of such proteins is directly associated with impaired bacterial growth and survival. Similarly, 3Q8U from *S. aureus* represents a well-characterized enzymatic target implicated in bacterial viability and pathogenicity. Targeting proteins from Gram-positive organisms is particularly important due to their clinical significance in food poisoning, skin infections, and systemic diseases^96,97^.

A more detailed analysis of ligand–protein interactions revealed that binding stability is governed not only by docking scores but also by the nature and number of interactions formed within the active site. The ligand and its metal complexes demonstrated key interactions, including hydrogen bonding with residues such as SER and GLU, as well as ionic and π-cation interactions with ARG and LYS residues. These interactions are known to enhance binding stability and may contribute to inhibition of protein function. Notably, some metal complexes exhibited multiple ionic interactions, which could strengthen electrostatic stabilization within the binding pocket despite having moderate docking scores.

The docking analysis indicates that coordination with transition metals enhances the binding efficiency of ligand and its complexes to bacterial proteins (Tables 7, 8, 9, 10 and Tables S2-S4). The Co(II) and UO_2_(II) complexes consistently exhibited stronger docking scores and more stable interactions, likely due to their ability to form multiple hydrogen bonds and ionic interactions (Tables 7, 8, 9, 10). The presence of π-cation interactions (notably in *S. aureus* and *S. typhi*) may contribute to stronger anchoring within the binding pocket, consistent with reports that π-interactions enhance docking stability of Schiff-base metal complexes^59,98^. Interestingly, the free ligand showed strong binding to *E. coli*, whereas metal incorporation improved binding in *S. aureus* and *S. typhi*, suggesting a pathogen-dependent effect of metal coordination. Cu(II) and Mn(II) complexes, while forming interactions, generally exhibited weaker binding energies, which may reflect lower stability or less favorable electronic compatibility with active-site residues. The observed interactions with catalytically essential residues (e.g., Ser209 in *B. cereus*, Arg102 in *S. aureus*, Arg91 in *E. coli*, Arg136 in *S. typhi*) suggest potential inhibition of bacterial enzymatic functions. These findings align with earlier reports where transition-metal complexes improved antimicrobial efficacy through enhanced protein–ligand interactions^16–19^. For Gram-negative pathogens, 3T88 from *E. coli* and 6J90 from *S. typhi* were selected because these proteins play critical roles in enzymatic pathways necessary for bacterial replication and survival^36^. Gram-negative bacteria possess an additional outer membrane that limits drug penetration; therefore, identifying compounds capable of forming stable interactions with essential intracellular proteins is crucial for predicting real antimicrobial effectiveness^99^.

**Table 7:** Docking scores and energies of ligand and its complexes with Crystal structure of *Bacillus cereus* (PDB ID 1FEZ).

Compound	Docking score kcal/mol )S(	rmsd_refine	E_conf	E_place	E_score1	E_refine	E_score2
Ligand	− 6.6997	1.2359052	− 60.7037	− 51.413	− 11.8644	− 29.4089	− 6.6997
Cu(II)	− 4.33815	2.3333426	− 199.789	− 9.83030	− 6.4898	− 14.9043	− 4.33815
Co(II)	− 5.29881	2.9495823	− 437.335	− 46.6289	− 1.90177	− 111.818	− 5.29881
Ni(II)	− 4.2385	2.8011343	− 654.966	− 92.4089	− 0.71437	− 48.3108	− 4.2385
Mn(II)	− 0.46018	2.2268453	− 699.269	− 13.1367	− 5.63332	− 52.9015	− 0.46018
UO_2_(II)	− 5.83284	1.9972301	− 1553.78	− 49.9857	− 6.33936	− 6.6944	− 5.83284

**Table 8:** Docking scores and energies of ligand and its complexes with crystal structure of *Staphylococcus aureus* (PDB ID 3Q8U).

Compound	Docking Score kcal/mol )S(	rmsd_refine	E_conf	E_place	E_score1	E_refine	E_score2
Ligand	− 5.9229	1.8171388	− 72.4748	− 53.4995	− 11.6115	− 26.3367	− 5.9229
Cu(II)	− 4.76027	1.6340342	− 231.3	− 80.5772	− 11.2773	− 25.9935	− 4.76027
Co(II)	− 5.64119	1.5515981	− 645.797	− 34.6968	− 12.7181	− 24.8068	− 5.64119
Ni(II)	− 4.98028	1.1222451	− 707.656	− 44.7613	− 12.3622	− 22.7125	− 4.98028
Mn(II)	− 5.46369	2.3896725	− 757.965	− 47.354	− 13.1369	− 31.8119	− 5.46369
UO_2_(II)	− 5.84179	1.4481082	− 1554.8	− 6.34267	− 13.403	− 23.4992	− 5.84179

**Table 9:** Docking scores and energies of ligand and its complexes with crystal structure of *Escherichia coli* (PDB ID 3T88).

Compound	Docking score kcal/mol )S(	rmsd_refine	E_conf	E_place	E_score1	E_refine	E_score2
Ligand	− 6.88541	2.1879144	− 66.6943	− 77.4794	− 12.847	− 38.8517	− 6.88541
Cu(II)	− 5.41498	2.4508934	− 236.912	− 33.4289	− 10.962	− 29.3314	− 5.41498
Co(II)	− 5.84831	2.318701	− 631.152	− 48.3852	− 12.1134	− 11.2426	− 5.84831
Ni(II)	− 5.44628	2.9199162	− 679.899	− 48.4466	− 9.17429	− 22.3167	− 5.44628
Mn(II)	− 5.37724	2.2523122	− 762.087	− 50.3731	− 12.6167	− 27.3411	− 5.37724
UO_2_(II)	− 5.64263	2.6605527	− 1534.63	− 27.4151	− 11.4011	− 18.4597	− 5.64263

**Table 10:** Docking scores and energies of ligand and its complexes with crystal structure of *Salmonella typhi* (PDB ID 6J90).

Compound	Docking Score kcal/mol )S(	rmsd_refine	E_conf	E_place	E_score1	E_refine	E_score2
Ligand	− 5.32063	1.2040076	− 70.5204	− 76.7965	− 14.9095	− 11.1107	− 5.32063
Cu(II)	− 4.10281	1.8825793	− 246.49	− 5.98028	− 8.80014	− 15.8057	− 4.10281
Co(II)	− 6.10275	2.3934622	− 651.591	− 16.3745	− 5.38412	− 25.9937	− 6.10275
Ni(II)	− 5.19669	1.3591352	− 699.305	35.6818	− 6.41459	− 21.6912	− 5.19669
Mn(II)	− 4.94038	1.882081	− 728.787	− 56.577	− 11.0506	− 17.0853	− 4.94038
UO_2_(II)	− 5.04151	2.2016013	− 1550.86	− 30.5473	− 8.22854	− 12.7018	− 5.04151

**Fig. 8:**
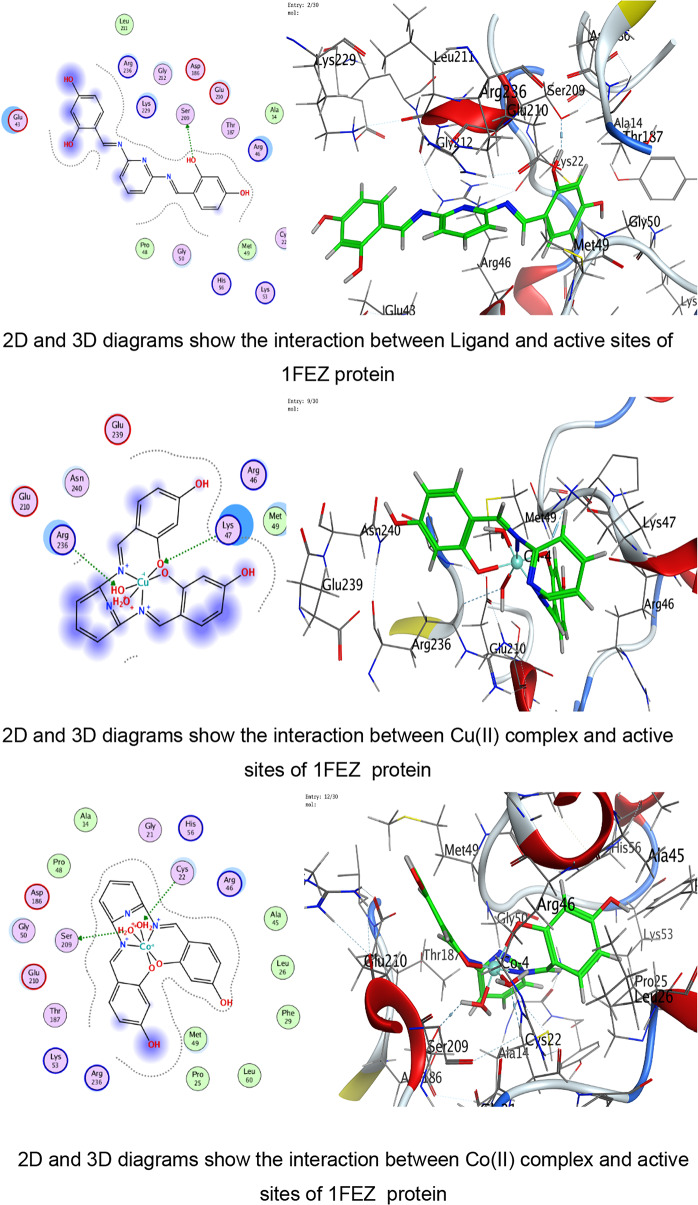

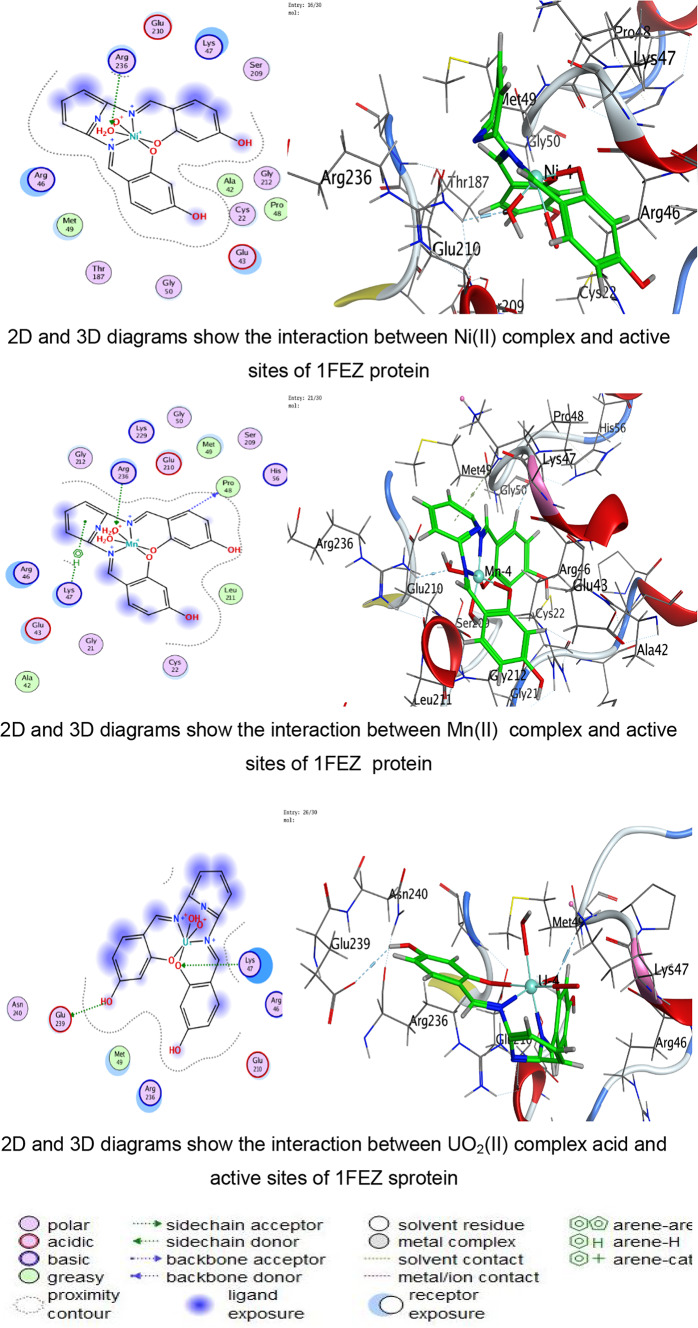
The representative key for the types of interaction between ligand and its metal complexes and *Bacillus cereus* PDB ID: 1FEZ protein.

**Fig. 9:**
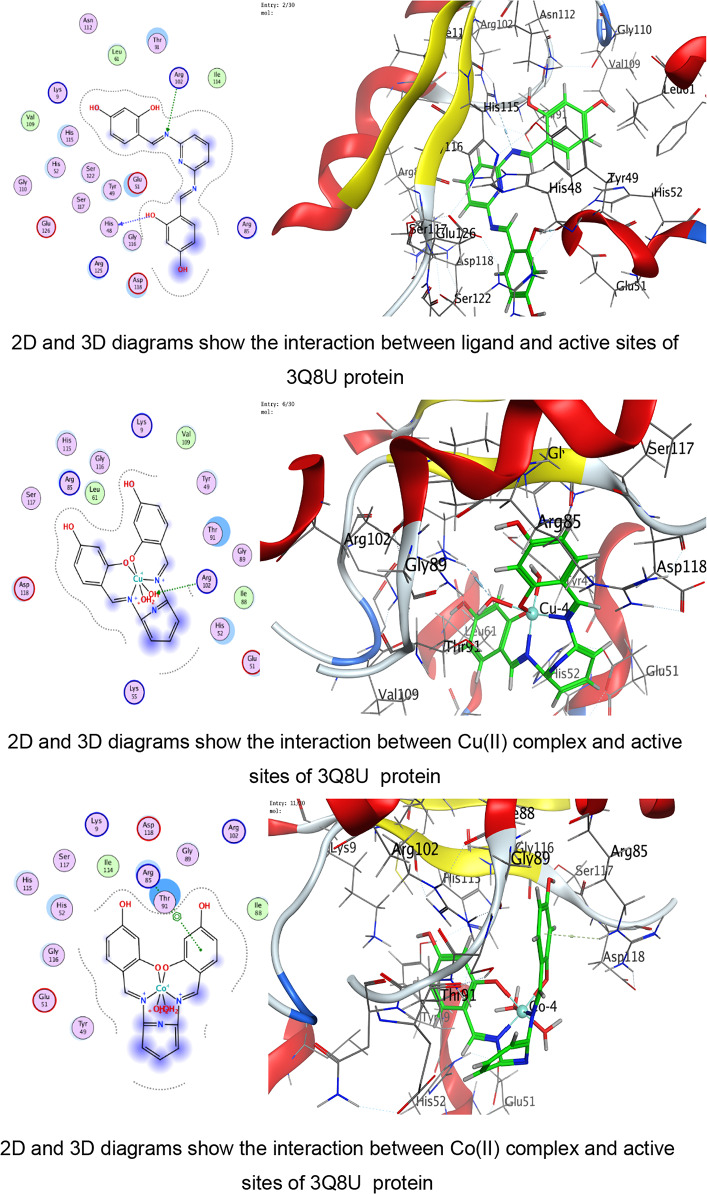

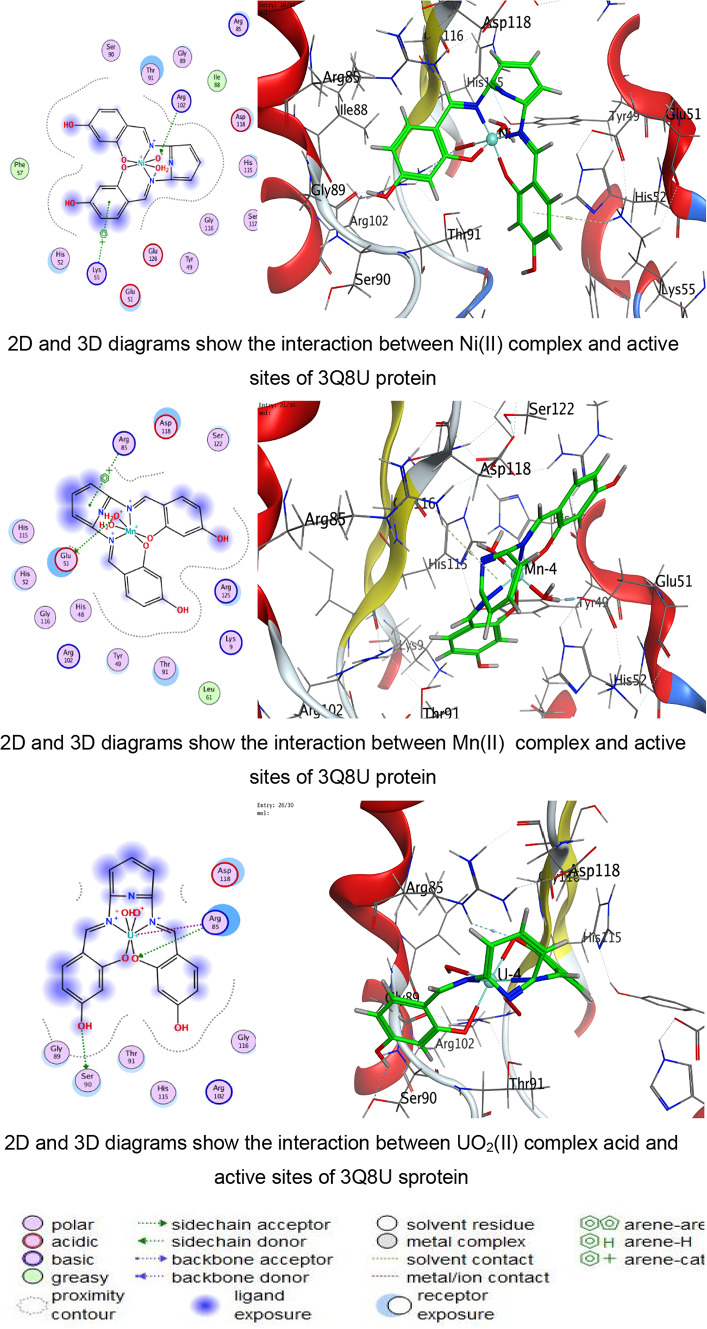
The representative key for the types of interaction between ligand and its metal complexes and *Staphylococcus aureus* PDB ID: 3Q8U protein.

**Fig. 10:**
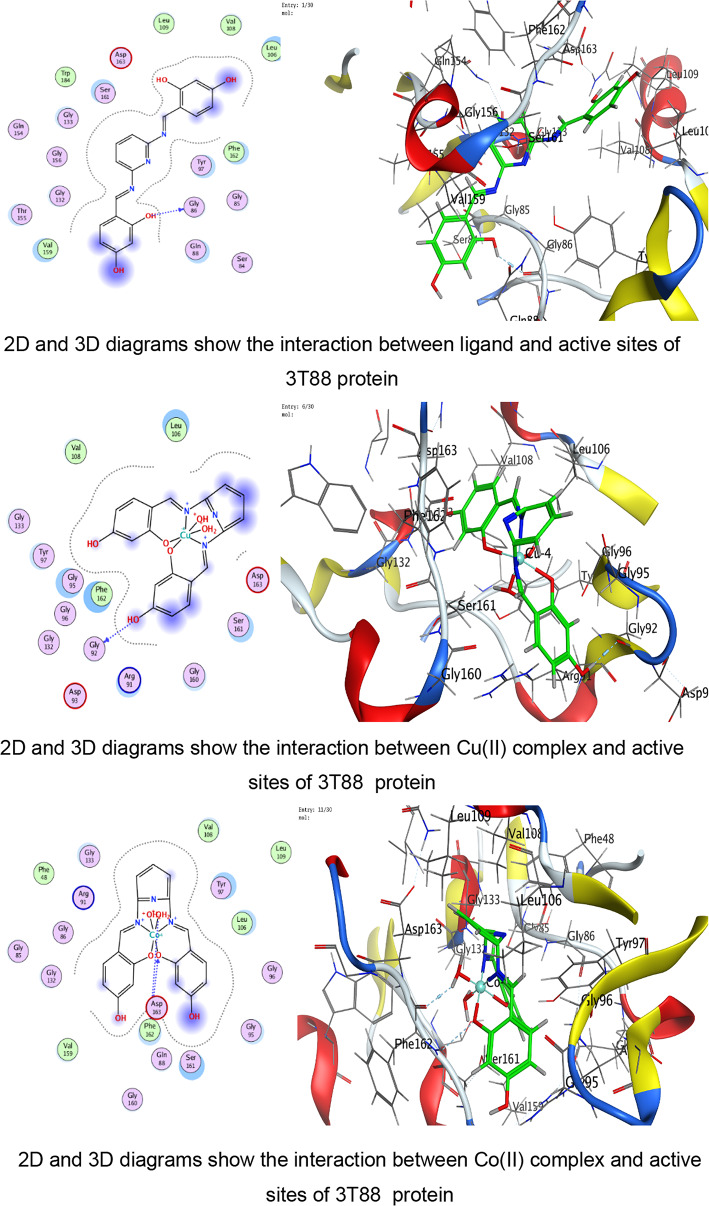

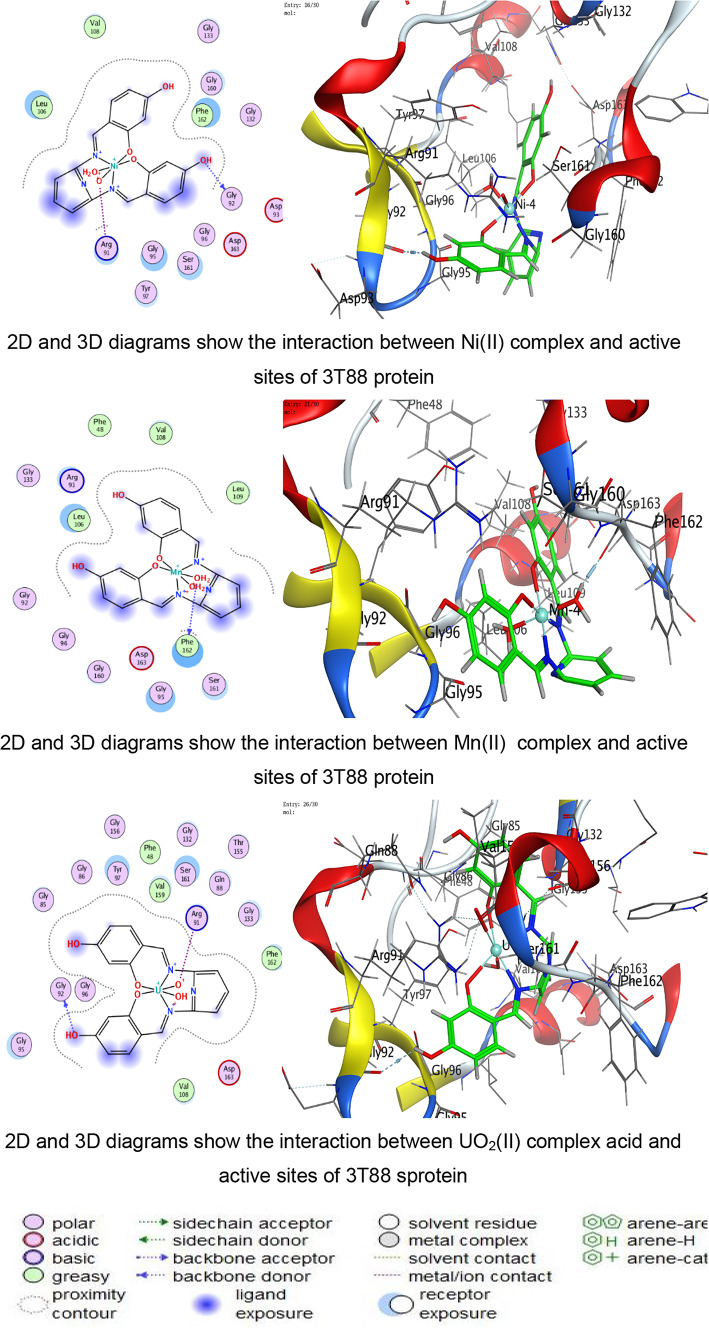
The representative key for the types of interaction between ligand and its metal complexes and *Escherichia coli* PDB ID:3T88 protein.

**Fig. 11:**
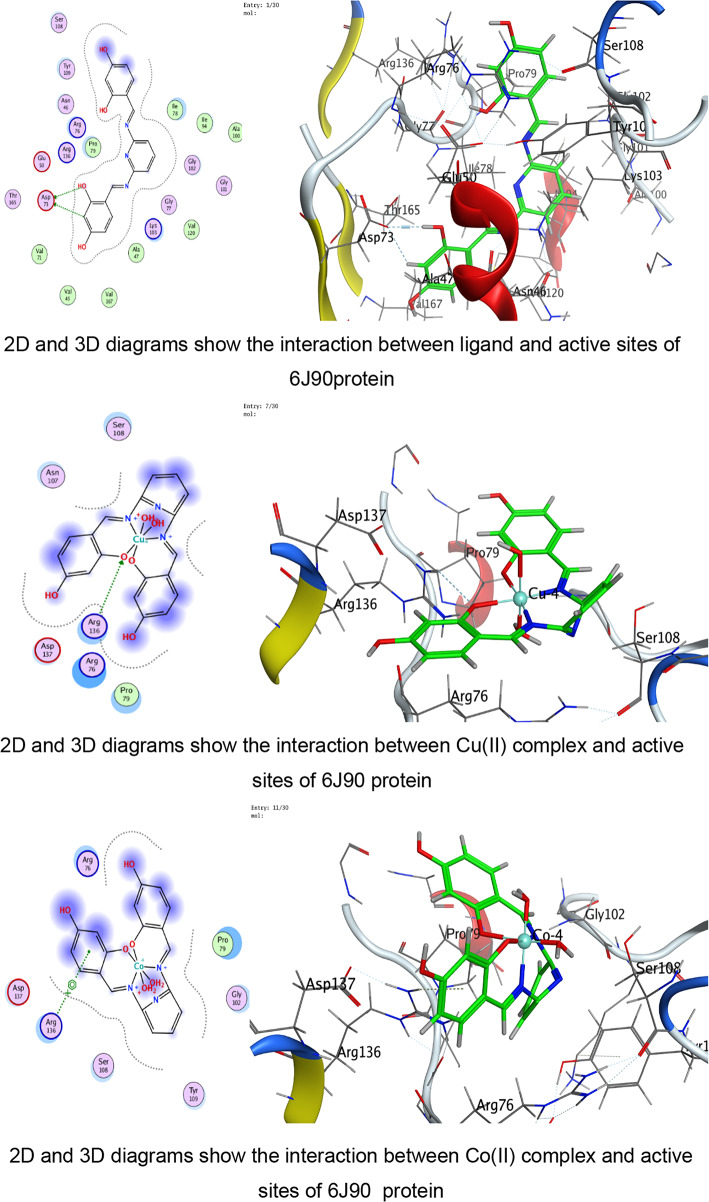

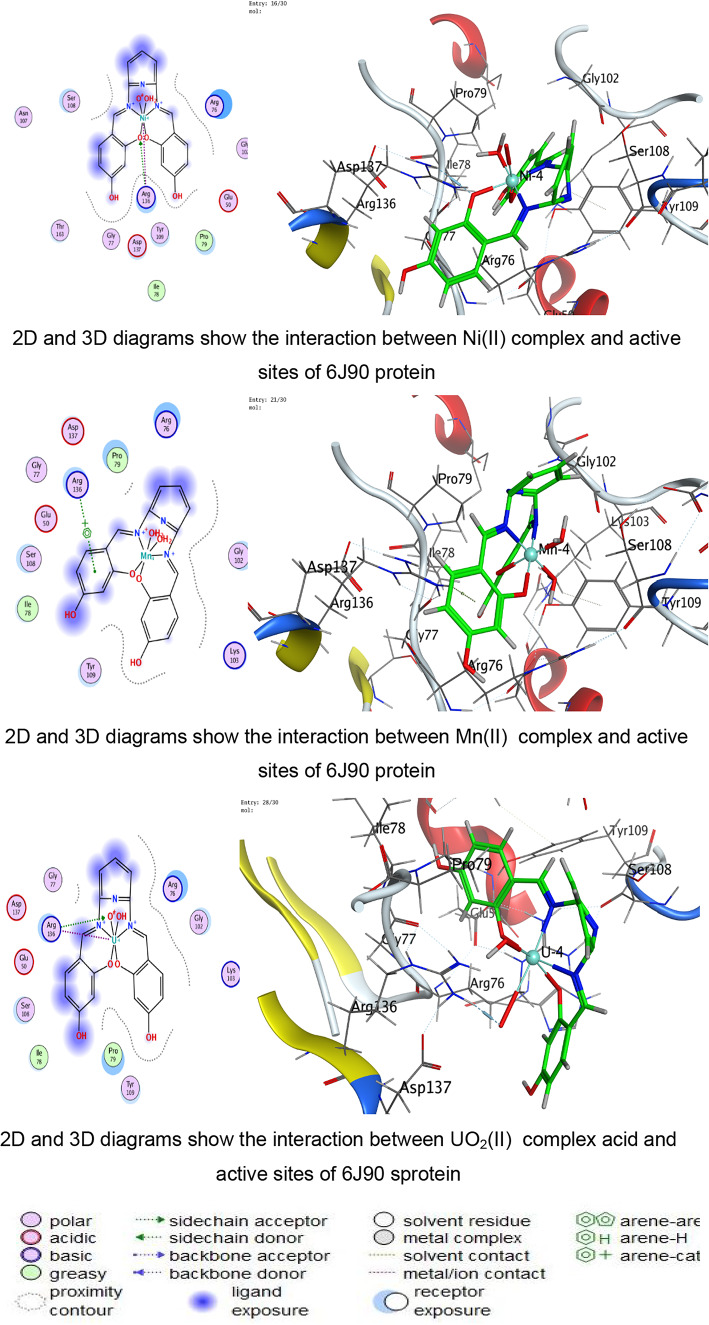
The representative key for the types of interaction between ligand and its metal complexes and *Salmonella typhi* PDB ID: 6J90 protein.

The use of experimentally resolved crystal structures with high resolution ensures structural accuracy, reliable binding-site definition, and meaningful docking predictions (Figs. 8, 9, 10, 11). For *B. cereus*, the free ligand exhibited the most favorable docking score (− 6.69 kcal/mol), followed by UO₂(II) (− 5.83 kcal/mol) and Co(II) (− 5.29 kcal/mol). Experimentally, however, UO₂(II) and Co(II) complexes showed enhanced antibacterial activity compared to several other complexes at higher concentrations. This slight divergence between docking score and biological activity suggests that, beyond binding affinity, physicochemical properties such as lipophilicity and membrane permeability contribute significantly to antimicrobial performance. Chelation theory explains this enhancement: coordination reduces metal ion polarity and increases lipophilicity, facilitating penetration through bacterial membranes^60,100^. This aligns with the improved ZOI observed for Co(II) and UO₂(II) complexes despite docking scores comparable to or slightly lower than the free ligand. For *S. aureus*, docking scores were relatively close among ligand (− 5.92), UO₂(II) (− 5.84), and Co(II) (− 5.64). Experimentally, UO₂(II) and Co(II) again demonstrated strong antibacterial effects. Interaction analysis revealed multiple hydrogen bonds and ionic interactions with ARG and SER residues, supporting stable binding within the active site. The presence of ionic and π-cation interactions in metal complexes likely enhances biological interference with enzymatic function^97^.

Against the Gram negative bacteria such as *E. coli*, the ligand showed the best docking score (− 6.88 kcal/mol), followed by Co(II) (− 5.84 kcal/mol) and UO₂(II) (− 5.64 kcal/mol). Experimentally, ZOI increased with concentration, and Co(II) and UO₂(II) complexes exhibited strong antibacterial effects. The reasonable agreement between docking affinity and antimicrobial activity supports the predictive value of the computational model. For *S. typhi*, Co(II) showed the best docking score (− 6.10 kcal/mol), correlating with its superior experimental ZOI. Notably, UO₂(II) formed multiple ionic interactions with ARG136, including strong hydrogen bonding (binding energy − 27.4 kcal/mol for one interaction), suggesting strong electrostatic stabilization. This explains its notable biological activity. The partial inconsistencies between docking rank and biological potency are expected because docking evaluates isolated protein–ligand affinity, whereas antimicrobial assays reflect multifactorial processes including cell wall penetration (especially critical in Gram-negative bacteria), metal ion dissociation and redox activity, ROS generation, and DNA/protein secondary interactions. In addition, metal complexes are known to exert antimicrobial activity through combined mechanisms beyond simple enzyme inhibition^68,69,97,100^.

However, the relationship between docking scores and experimental antimicrobial activity was not strictly linear. While certain complexes, such as Co(II) and UO₂(II), showed reasonable agreement between predicted binding affinity and biological activity, others demonstrated discrepancies. This observation highlights the limitations of docking, which does not account for important biological factors such as membrane permeability, solvation effects, protein flexibility, and intracellular mechanisms. Therefore, the docking results are better interpreted as providing qualitative mechanistic insights into ligand–protein interactions rather than establishing a direct quantitative correlation with antimicrobial activity. This interpretation is consistent with current best practices in molecular docking studies and supports the complementary use of computational and experimental approaches.

### Comparative analysis and novelty statement

The novelty of the present work is established through a multi-dimensional approach that distinguishes it from existing reports on Schiff base metal complexes. While this class of compounds is widely studied, the current research utilizes a novel tetradentate dibasic (N_2_O_2_) Schiff base ligand specifically derived from 2,6-diaminopyridine and 2,4-dihydroxybenzaldehyde. The manuscript highlights that diaminopyridines remain under-explored as ligand precursors compared to other diamines. Furthermore, this study extends beyond standard transition metals to include the UO_2_(II) complex, which demonstrated superior antifungal efficacy and unique binding stabilization in docking simulations.

Unlike many studies that focus solely on ZOI assays, the obtained biological profiling includes antibiofilm activities and intracellular enzymatic assays (POX and CAT), providing a deeper mechanistic understanding of how these complexes disrupt microbial survival. Additionally, the computational study targets four distinct bacterial proteins (PDB IDs: 1FEZ, 3Q8U, 3T88, and 6J90), ensuring a robust correlation between experimental bioactivity and molecular-level interactions across diverse pathogens. Tables 11 and 12 summarizes these key differentiators in comparison with recent literature.

**Table 11:** Novelty of current study vs. recent literature.

Ligand core/type	Metal complexes	Biological activities evaluated	Docking targets	References
4-Hydroxy-3-methoxybenzaldehyde + ethylenediamine (Bidentate)	Cu(II), Co(II), Ni(II), Zr(IV)	Antimicrobial, antioxidant (DPPH)	Not reported	^101^
3-(2-Furyl)acrolein + 2-amino-6-ethoxybenzothiazole	Lanthanides (Gd, Sm, Nd)	Antimicrobial, antitumor, antioxidant	Not reported	^102,103^
General pyridine Schiff bases	General transition Metals	General antimicrobial review	Not reported	^97,104^
2,6-Diaminopyridine + benzaldehyde (bidentate)	Co(II), Ni(II), Cu(II)	Antimicrobial	Not reported	^105^
2,6-Diaminopyridine + 2,4-dihydroxybenzaldehyde (tetradentate)	Cu(II), Co(II), Ni(II), Mn(II), UO_2_(II)	Antimicrobial, antibiofilm, antioxidant, POX/CAT enzymatic assays	4 Targets (1FEZ, 3Q8U, 3T88, 6J90)	This study

**Table 12:** Comparative analysis of the current study vs. existing literature on Schiff base complexes.

Feature	Recent literature	This study
Ligand type	Often bidentate or common scaffolds^106^	Novel tetradentate (N_2_O_2_) dibasic ligand
Metal centers	Primarily 1st row transition metals (Cu, Co, Ni)^107,108^	Cu, Co, Ni, Mn plus UO_2_(II)
Bio-evaluation	Standard antimicrobial (ZOI/MIC)^104,107–109^	Antimicrobial + antibiofilm + POX/CAT enzymes
Antioxidant	General DPPH screening^74,92^	High DPPH (78.1% for Mn) + enzyme correlation
Docking study	Limited targets or absent^107,108,110,111^	Multi-target (4 PDBs) with 2D/3D interaction profiling
Efficacy trend	Complex > ligand^102,103,105–112^	Validated complex > ligand with mechanistic proof

## Conclusion

The ligand and its complexes were produced by interacting with Cu(II), Co(II), Ni(II), Mn(II), and UO_2_(II) acetates. The structures were characterized using the ligand and its complexes’ elemental analysis, FT-IR, magnetic moment, molar conductance, XRD, and FT-IR spectra data. Numerous techniques have been used to characterize the compounds. Schiff base plays a tetradentate dibasic donor by coordinating across azomethine nitrogen and phenolic –O groups. Ligand and complexes shown talented binding affinities for therapeutically relevant pathogens. Co(II) and UO_2_(II) complexes were the most successful candidates; they established persistent hydrogen bonds with significant residues as well as π-cation contacts and ionic interactions. Moreover, the present study demonstrates a clear relationship between computational predictions and experimental antimicrobial performance of the investigated ligand and its metal complexes. Molecular docking against essential bacterial proteins from *B. cereus*, *S. aureus, E. coli*, and *S. typhi* revealed favorable binding affinities, particularly for Co(II) and UO₂(II) complexes. These theoretical findings were largely supported by in vitro antimicrobial assays, where the same complexes exhibited enhanced ZOI compared to the free ligand and several other metal derivatives. The study confirms that metal coordination improves biological activity, likely through increased lipophilicity, stronger electrostatic interactions within protein active sites, and potential multi-target effects beyond single-enzyme inhibition. While docking provided valuable insight into binding stability and interaction patterns, experimental results highlighted the additional contribution of physicochemical and cellular factors such as membrane permeability and metal-mediated oxidative mechanisms. The partial agreement observed between docking predictions and experimental results supports the role of molecular docking as a complementary tool for mechanistic interpretation, while also highlighting its inherent limitations. This study provides a foundation for the rational design of metal-based antimicrobial agents. The investigated compounds may serve as promising lead structures for further optimization. Future work should focus on in vivo validation, toxicity assessment, pharmacokinetic studies, and advanced computational analyses such as molecular dynamics simulations to better understand stability, bioavailability, and mechanism of action under physiological conditions.

## Supplementary Information

Below is the link to the electronic supplementary material.

Supplementary Material 1

## Data Availability

Data for experimental runs are presented in this paper and the supplementary information. The datasets utilized and/or examined in the present investigation are obtainable from the relevant author upon reasonable request.
