# Supplementary material for: Antimicrobial, antibiofilm, antioxidant activities and molecular docking of Schiff base and its complexes of Cu(II), Co(II), Ni(II), Mn(II) and UO2(II)
Source: Sci Rep. 2026 Jun 5;16:17519. doi: 10.1038/s41598-026-54902-5 (PMC13241536; doi:10.1038/s41598-026-54902-5)
Supplement: Supplementary file 1 — Supplementary Material 1 [file 41598_2026_54902_MOESM1_ESM.docx]

**Supplementary Tables**

**Table S1**. TGA thermal data of ligand and its complexes **(1-5)**.

| **Compound** | **Temperature range (°C)** | **TG weight loss %** | | **Assignments** |
| --- | --- | --- | --- | --- |
|  |  | **Found** | **Calc.** |  |
| **Ligand** | 60-143 | 4.92 | 4.87 | OH |
|  | 143-210 | 7.62 | 7.16 | C_2_H |
|  | 210-590 | 85.73 | 86.25 | C_16.5_H_13_N_3_O_3_ |
|  | < 800 | 1.73 | 1.72 | 0.5C |
|  |  |  |  |  |
| **(1)** | 130-440 | 31.16 | 31.35 | 2H_2_O + C_7_H_4_O |
|  | 440-587 | 48.88 | 48.15 | C_11_H_9_N_3_O_2_ |
|  | Remain | 19.96 | 20.50 | CuO + C |
|  |  |  |  |  |
| **(2)** | 46-115 | 7.98 | 7.53 | 2H_2_O |
|  | 115-540 | 68.60 | 69.26 | 2H_2_O +C_16_H_13_N_3_O_3_ |
|  | Remain | 23.42 | 23.21 | CoO + 3C |
|  |  |  |  |  |
| **(3)** | 49-100 | 10.63 | 10.89 | 3H_2_O |
|  | 100-503 | 71.38 | 71.61 | 2H_2_O +C_18_H_13_N_3_O_3_ |
|  | Remain | 17.98 | 17.49 | NiO + C |
|  |  |  |  |  |
| **(4)** | 49-107 | 14.10 | 14.12 | 4H_2_O |
|  | 107-558 | 69.36 | 69.61 | 2H_2_O +C_18_H_13_N_3_O_3_ |
|  | Remain | 16.54 | 16.27 | MnO + C |
|  |  |  |  |  |
| **(5)** | 46-100 | 8.06 | 8.05 | 3H_2_O |
|  | 100-400 | 32.46 | 31.60 | C_12_H_8_N_2_O_2_ |
|  | 400-520 | 19.36 | 20.12 | C_7_H_5_NO_2_ |
|  | Remain | 40.12 | 40.23 | UO_2_ |

^a^Numbers as given in Fig. 1.

**Table S2.** Interaction of ligand and its complexes with Crystal Structure of Bacillus cereus (PDB ID 1FEZ).

| **Compound** | **Atom Involved** | **Residue** | **Interaction Type** | **Distance (Å)** | **Binding Energy (kcal/mol)** |
| --- | --- | --- | --- | --- | --- |
| Ligand | O 21 | OG SER 209 (A) | H-donor | 2.75 | -2.0 |
| Cu(II) | O 22 | NZ LYS 47 (A) | H-acceptor | 2.99 | -5.6 |
|  | O 40 | CD ARG 236 (A) | H-acceptor | 3.40 | -0.5 |
| Co(II) | O 40 | OG SER 209 (A) | H-donor | 2.79 | -0.9 |
|  | O 43 | CB CYS 22 (A) | H-acceptor | 3.0 | -0.6 |
| Ni(II) | O 41 | NH1 ARG 236 (A) | H-acceptor | 2.76 | -2.7 |
| Mn(II) | C 17 | O PRO 48 (A) | H-donor | 3.09 | -0.5 |
|  | O 40 | NH1 ARG 236 (A) | H-acceptor | 2.61 | -1.2 |
|  | 6-ring | CD LYS 47 (A) | pi-H | 4.44 | -0.7 |
| UO_2_(II) | O 23 | OE2 GLU 239 (A) | H-donor | 3.24 | -1.5 |
|  | O 21 | NZ LYS 47 (A) | H-acceptor | 3.05 | -5.7 |

**Table S3.** Interaction of ligand and its complexes with Crystal Structure of Staphylococcus aureus (PDB ID 3Q8U).

| **Compound** | **Atom Involved** | **Residue** | **Interaction Type** | **Distance (Å)** | **Binding Energy (kcal/mol)** |
| --- | --- | --- | --- | --- | --- |
| Ligand | O 23 | O HIS 48 (A) | H-donor | 3.29 | -1.3 |
|  | N 27 | NH2 ARG 102 (A) | H-acceptor | 3.66 | -1.5 |
| Cu(II) | O 40 | NH2 ARG 102 (A) | H-acceptor | 3.25 | -4.6 |
| Co(II) | 6-ring | NE ARG 85 (A) | pi-cation | 3.84 | -1.3 |
| Ni(II) | O 40 | NH1 ARG 102 (A) | H-acceptor | 3.59 | -0.6 |
|  | O 40 | NH2 ARG 102 (A) | H-acceptor | 3.29 | -12.8 |
|  | O 40 | NH1 ARG 102 (A) | Ionic | 3.59 | -1.6 |
|  | O 40 | NH2 ARG 102 (A) | Ionic | 3.29 | -2.8 |
|  | 6-ring | NZ LYS 55 (A) | pi-cation | 4.57 | -1.5 |
| Mn(II) | O 40 | OE2 GLU 51 (A) | H-donor | 2.70 | -4.6 |
|  | 6-ring | NH1 ARG 85 (A) | pi-cation | 4.42 | -1.1 |
| UO_2_(II) | O 23 | OG SER 90 (A) | H-donor | 3.02 | -0.5 |
|  | O 21 | NE ARG 85 (A) | H-acceptor | 3.06 | -0.6 |
|  | U 40 | NE ARG 85 (A) | Ionic | 3.44 | -2.1 |
|  | U 40 | NH1 ARG 85 (A) | Ionic | 3.54 | -1.7 |

**Table S4.** Interaction of ligand and its complexes with Crystal Structure of Escherichia coli (PDB ID 3T88).

| **Compound** | **Atom Involved** | **Residue** | **Interaction Type** | **Distance (Å)** | **Binding Energy (kcal/mol)** |
| --- | --- | --- | --- | --- | --- |
| Ligand | O 23 | O GLY 86 (A) | H-donor | 2.78 | -1.9 |
| Cu(II) | O 23 | O GLY 92 (A) | H-donor | 2.81 | -2.0 |
| Co(II) | O 43 | O PHE 162 (A) | H-donor | 2.70 | -1.1 |
|  | O 22 | N PHE 162 (A) | H-acceptor | 2.96 | -0.5 |
| Ni(II) | O 23 | O GLY 92 (A) | H-donor | 2.79 | -2.2 |
|  | O 40 | NE ARG 91 (A) | Ionic | 3.39 | -2.4 |
|  | O 40 | NH2 ARG 91 (A) | Ionic | 3.26 | -3.0 |
| Mn(II) | O 43 | O PHE 162 (A) | H-donor | 2.79 | -4.2 |
| UO_2_(II) | O 19 | O GLY 92 (A) | H-donor | 3.09 | -0.7 |
|  | O 41 | NH1 ARG 91 (A) | Ionic | 3.31 | -2.7 |

**Supplementary Figures**

|  | ***S. aureus*** | ***S. typhi*** | ***C. albicans*** | ***A. niger*** | ***F. oxysporum*** | ***Penicillium* sp.** |
| --- | --- | --- | --- | --- | --- | --- |
| **Ligand** | 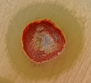 | 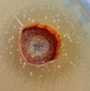 | 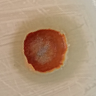 | 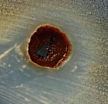 | 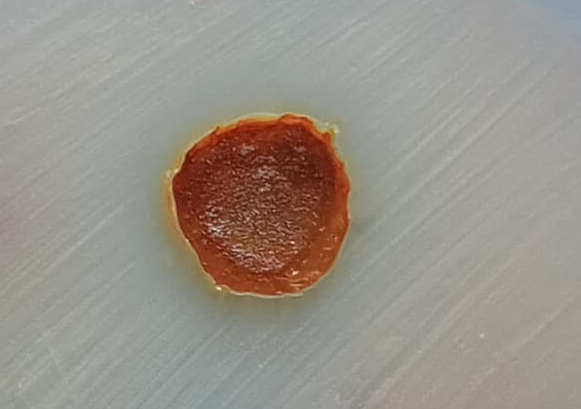 | 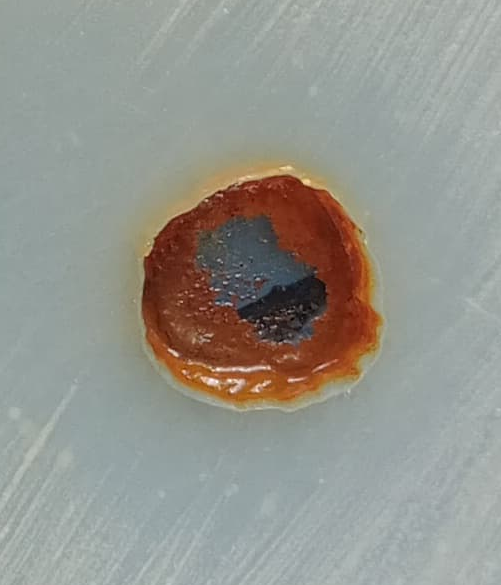 |
| **Cu(II)** | 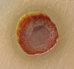 | 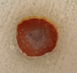 | 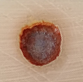 | 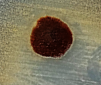 | 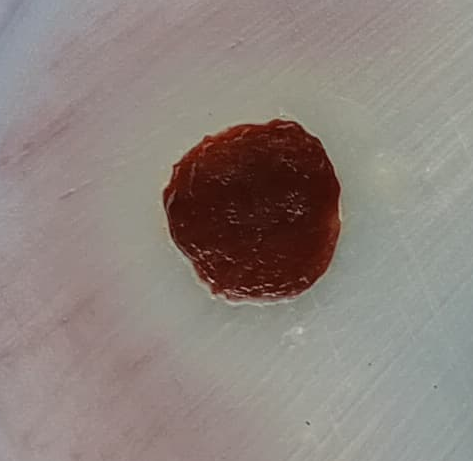 | 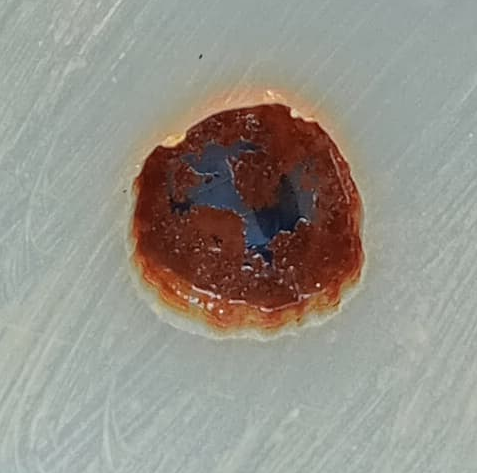 |
| **Co(II)** | 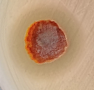 | 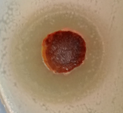 | 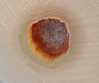 | 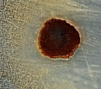 | 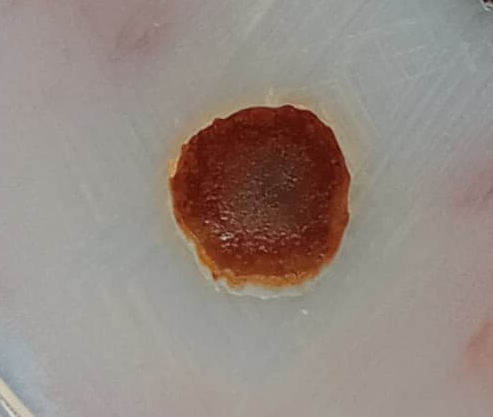 | 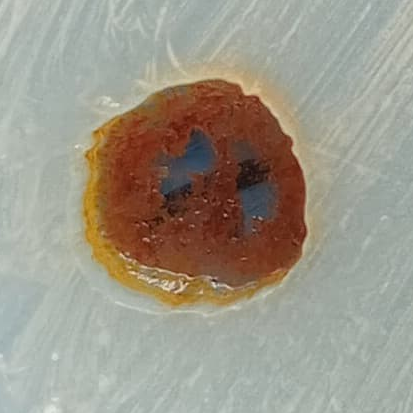 |
| **Ni(II)** | 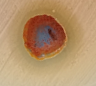 | 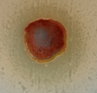 | 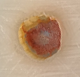 | 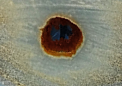 | 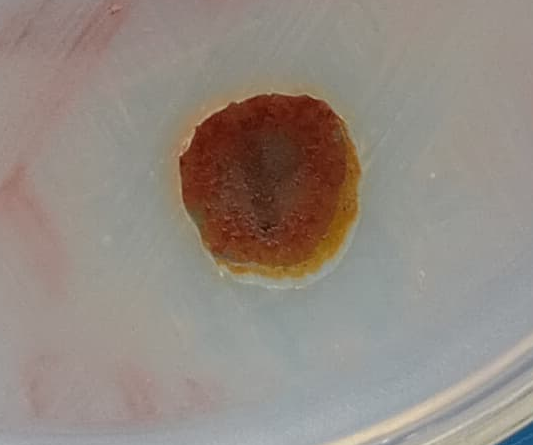 | 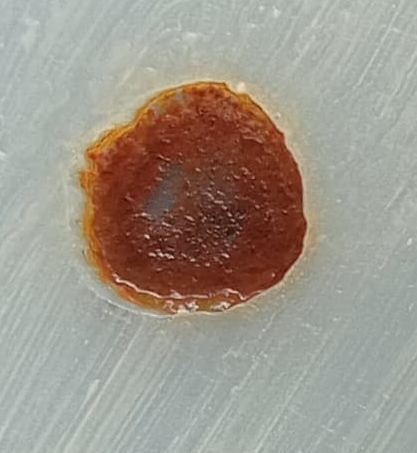 |
| **Mn(II)** | 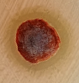 | 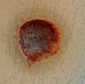 | 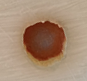 | 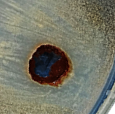 | 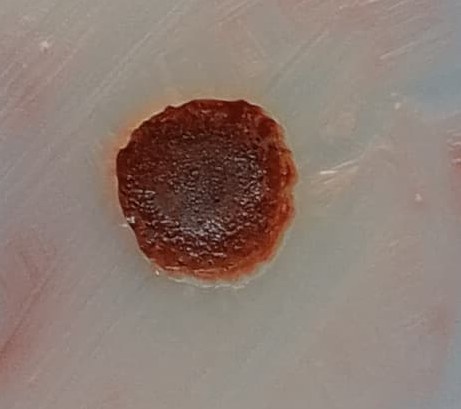 | 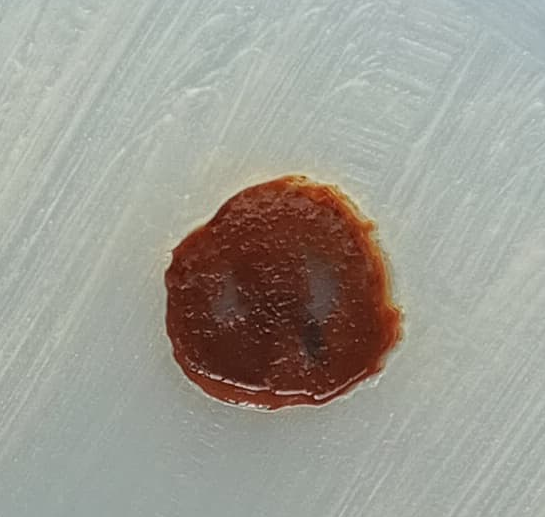 |
| **UO_2_(II)** | 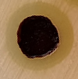 | 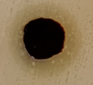 | 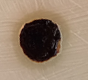 | 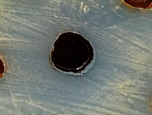 | 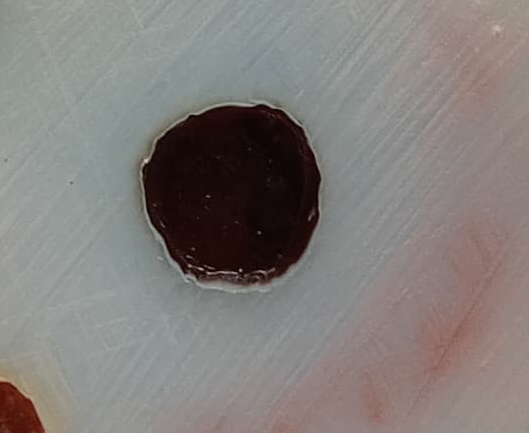 | 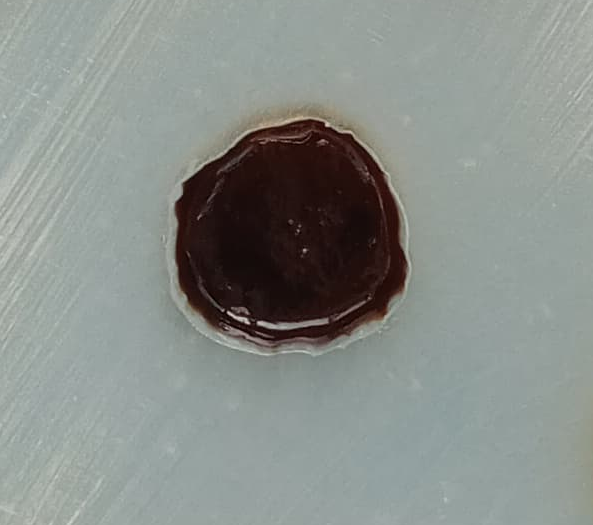 |

**Fig. S1.** Visual evaluation of the antimicrobial and antifungal efficacy via the agar well diffusion method. The figure displays the representative inhibition plates for the Schiff base ligand and its metal complexes (Cu(II), Co(II), Ni(II), Mn(II), and UO_2_(II)) against bacterial pathogens (*S. aureus* and *S. typhi*) and fungal strains (*C. albicans*, *A. niger*, *F. oxysporum*, and *Penicillium* sp.).
